# A multi-antigen vaccinia vaccine broadly protected mice against SARS-CoV-2 and influenza A virus while also targeting SARS-CoV-1 and MERS-CoV

**DOI:** 10.3389/fimmu.2024.1473428

**Published:** 2024-11-28

**Authors:** Nan Gao, Tianhan Yang, Lanlan Dong, Wanda Tang, Kangli Cao, Longfei Ding, Cuisong Zhu, Shimeng Bai, Ai Xia, Youwei Zhu, Chen Zhao, Haoran Peng, Jianqing Xu, Xiaoyan Zhang

**Affiliations:** ^1^ Shanghai Public Health Clinical Center and Institutes of Biomedical Sciences, Fudan University, Shanghai, China; ^2^ Department of Microbiology, Second Military Medical University, Shanghai, China; ^3^ Clinical Center of Biotherapy at Zhongshan Hospital and Shanghai Institute of Infectious Disease and Biosecurity, Fudan University, Shanghai, China; ^4^ Clinical Center for Biotherapy, Zhongshan Hospital (Xiamen), Fudan University, Xiamen, Fujian, China; ^5^ Bio-therapeutic Center, National Clinical Research Center for Infectious Disease, Shenzhen Third People’s Hospital, The Second Hospital Affiliated with the School of Medicine, Southern University of Science and Technology, Shenzhen, Guangdong, China; ^6^ Xiamen Key Laboratory of Biotherapy, Xiamen, Fujian, China

**Keywords:** coronavirus, SARS-CoV-2, influenza virus, vaccinia virus Tiantan strain, multipathogen vaccine, mucosal immunity

## Abstract

**Introduction:**

Coronaviruses and influenza viruses are significant respiratory pathogens that cause severe disease burdens and economic losses for society. Due to their diversity and evolution, vaccines typically require periodic updating to remain effective. An additional challenge is imposed by the possible coinfection of SARS-CoV-2 and influenza, which could increase disease severity.

**Methods:**

We developed a vaccinia vaccine, named rTTV-RBD-HA2, broadly targeting coronaviruses and influenza viruses. This vaccine expresses three fusion proteins, each comprising the receptor-binding domain (RBD) from one of the three highly pathogenic coronaviruses (SARS-CoV-2, SARS-CoV, and MERS-CoV) and the conserved HA stalk region from two influenza viruses (pdmH1N1 and nH7N9) belonging to groups 1 and 2, respectively.

**Results:**

The multi-targeting nature of this vaccine was validated by its success in inducing antibody responses to the three RBDs and both group 1 and 2 HAs in mice. Importantly, it also generated robust T cell responses to all the immunogens, which could be mobilized to the lung through intranasal vaccination. Consistent with this broad immunogenicity profile, when administered via intramuscular priming and two intranasal boosts, rTTV-RBD-HA2 effectively protected vaccinated mice against challenges of the wild-type SARS-CoV-2 virus, the Omicron XBB variant, and the influenza A H1N1 and H3N2 viruses.

**Discussion:**

Our results collectively support the candidacy of recombinant rTTV-RBD-HA2 as a novel respiratory virus vaccine that provides cross-protection against coronaviruses and influenza viruses, surpassing the breadth of previous vaccines. Additionally, they underscore the importance of establishing a strong mucosal T cell response in the development of a universal respiratory virus vaccine.

## 1 Introduction

Since its appearance in 2019, severe acute respiratory syndrome coronavirus 2 (SARS-CoV-2) has caused over 760 million infections (1). Its threat to humans persists as mutant variants continue to emerge with an increasing ability to evade the immuno-protection, particularly antibody responses, established through prior vaccination or infection with earlier strains of the virus. Also needed to be on the alert are other zoonotic coronaviruses that caused local outbreaks in the past, including severe acute respiratory syndrome coronavirus (SARS-CoV) and Middle East respiratory syndrome coronavirus (MERS-CoV), of which the latter still has sporadic confirmed cases (2, 3).

Compared to the coronavirus, influenza A and B viruses posed even more serious challenges in causing respiratory infections (4). The threats come from seasonal epidemics of both viruses and pandemics of influenza A virus that occur upon cross-species spillover (5). The annual number of cases of seasonal influenza is estimated to be around 1 billion, with 3 to 5 million being severe and associated with 290000 to 650000 deaths (6). Influenza A viruses have caused four pandemics in 1918, 1957, 1968, and 2009. Among these, the 1918 H1N1 pandemic is the worst, with the highest mortality rate and resulting in a global death toll of approximately 50 million (7). Of note, the 2009 pandemic, caused by an H1N1 virus (pdmH1N1) of swine origin, prompted a profound public response that included the development of the tailored vaccines. The next influenza A virus with pandemic potential was exemplified by the avian H7N9 strain (nH7N9) first identified in 2013. As of January 31, 2024, the total number of laboratory-confirmed human cases of nH7N9 infection had reached 1568, among which 616 were fatal cases, equaling a mortality rate of approximately 39 percent (8). Despite epidemiological data indicating that the current nH7N9 virus is unlikely to cause an influenza pandemic, its animal-to-human spillover and its potential for gaining human-to-human transmission capability need regular monitoring with vigilance (9). The influenza virus vaccine program is widely considered as one of the most challenging due to its RNA polymerase being more error-prone than that of the coronavirus and its segmented genome allowing for virus reassortment in co-infecting cells. The predominantly deployed vaccine, the trivalent or quadrivalent inactivated vaccine, needs to be administered annually, with efficacy varying unpredictably depending on how well the included strains match the actually circulating ones.

Though challenging due to the divergence and evolution of viruses, as mentioned above, vaccination is considered the only measure that could achieve herd immunity against the coronaviruses and influenza viruses. Most developed vaccines against the two viruses have been based on inducing a virus-specific antibody response, with the Spike (S) protein and hemagglutinin (HA) protein as the primary targets for coronaviruses and influenza viruses, respectively. Both proteins mediate viral entry by binding corresponding receptors on cell surfaces. The antibody response specific to the S protein induced by viral infection or vaccination is predominantly directed against its receptor-binding domain (RBD), which is the target of most neutralizing antibodies. This rationalizes using RBD instead of full-length S-protein as the immunogen in many vaccines, to avoid the production of non-neutralizing antibody to protein sequences outside of the RBD that might have adverse effects (10–12). Compared to HA2, HA1 is immunodominant and is the main target of neutralizing antibodies. However, HA1 has a lower level of conservation and can tolerate mutations that disrupt the neutralizing epitopes. Such plasticity is a primary challenge for vaccines using the whole HA protein as the immunogen. On the other hand, HA2-specific antibodies, which can be induced by vaccines expressing only HA2 or strategies designed to subvert the immunodominance of HA1, have a broader cross-reactivity than antibodies against HA1. They are capable of providing cross-subtype and even cross-group protection by blocking fusion peptide activity or eliminating infected host cells through antibody-dependent cell-mediated cytotoxicity (ADCC) (13–15). This substantiates HA2 as a promising immunogen for developing a universal influenza virus vaccine.

Besides the immunogen, the type of vaccine and its administration route are also determining factors of the efficacy of protection. Specifically, with the same immunogen, vaccines of different types could elicit immune responses varying in involvement of antibodies and T-cell immunity. Leveraging a balanced humoral and cellular immunity is considered an optimal characteristic of a potent vaccine (4, 16). This characteristic is well embodied in viral vector vaccines, which are advantageous in eliciting an immune response similar to natural infection and capable of establishing mucosal immunity through the appropriate mode of administration (17). In the history of viral vector vaccines, the vaccinia virus (VV) is a long-standing one, known for its use as a live vaccine to eradicate smallpox. As an enveloped, double-stranded DNA virus belonging to the family Poxviradae, VV is relatively safe because, with its entire life cycle occurring exclusively in the cytoplasm, there is no integration of viral sequence into the genome of the infected host cell (18). The promise of VV as a vaccine vector also aligns with its large genome size, which allows for the insertion of DNA sequences encoding large immunogens or multiple immunogens in the same vector. It is worth noting that there was a regional difference in the traditional VV strains exploited as the smallpox vaccine. The Tiantan strain, TTV-752-1, is the strain used in China, and its utility in generating vaccines targeting various viruses has been extensively studied (19–21).

In addition to the aforementioned continuing threats individually posed by the coronavirus and influenza virus, there is a growing concern that these two viruses could co-infect individuals as they circulate within the same population. This co-infection may result in clinical outcomes that differ from those of individual infections (22, 23). Therefore, developing a vaccine that can effectively target both viruses would be desirable. In pursuit of this goal, we recently developed a dual-targeting immunogen design by combining RBD of the SARS-CoV-2 with the HA2 of the H7N9 influenza virus as a fusion moiety. Using mouse infection models, we demonstrated that a chimpanzee adenovirus 68 (AdC68)-based vaccine expressing this immunogen provided effective protection against lethal challenges of both SARS-CoV-2 and H7N9 viruses, as well as morbidity induced by H3N2 influenza virus (24). In this study, we aimed to explore further this design for generating a vaccine with a more expanded spectrum against coronaviruses and influenza viruses. Specifically, we leveraged the large coding capacity of TTV-752-1 VV to express three synthetic RBD-HA2 immunogens that contain RBD from SARS-CoV-2, SARS-CoV, and MERS-CoV in combination with HA2 from the H7N9 virus causing most recent human infection (nH7N9) or the H1N1 virus underlying the 2009 H1N1 pandemic (pdmH1N1). The thus constructed vaccine, rTTV-RBD-HA2, demonstrated a strong ability to induce neutralizing antibodies against the three targeted coronaviruses and influenza viruses from both HA groups. Moreover, when given in a regimen that included an intramuscular prime followed by intranasal boosting, it was able to elicit broad and potent virus-specific T-cell responses at mucosal sites. This effect could not be achieved through intramuscular vaccinations alone. Importantly, the intramuscular-intranasal regimen showed superiority over the intramuscular-only regimen in protective efficacy against both SARS-CoV-2 (wild-type and the Omicron variant) and cross-group subtype influenza viruses (pdmH1N1 and H3N2, respectively), consistent with a critical contribution of respiratory mucosal immunity to protection. Our study supported the candidacy of rTTV-RBD-HA2 as a pan-vaccine to prevent respiratory diseases caused by both coronaviruses and influenza viruses. It also suggested that, in order to be effective, such a pan-vaccine might need to prioritize the induction of a strong mucosal T-cell response in addition to neutralizing antibodies.

## 2 Materials and methods

### 2.1 Cell lines

The human embryonic kidney (HEK) 293T cell line, human osteosarcoma cell line 143B, human hepatoma cell line Huh-7, and Madin-Darby canine kidney (MDCK) cell line were obtained from the American Type Culture Collection (ATCC; Manassas, USA). The HEK293T cell line stably expressing human angiotensin-converting enzyme 2 (hACE2), hACE2-293T, was generated in-house as previously described (25). The chicken fibroblast cell line DF-1 was generously provided by Dr. Zhengfan Jiang (Peking University, Beijing, China). All of these cell lines were maintained in complete Dulbecco’s modified Eagle’s medium (DMEM; Corning, New York, USA) supplemented with 10% fetal bovine serum (FBS; BI, Kibbutz Beit Haemek, Israel) and 1% penicillin-streptomycin (PS; Gibco, New York, USA) at 37°C in a humidified incubator with 5% CO_2_.

### 2.2 Viruses

The Tiantan vaccinia virus (TTV-752-1) and the A/California/04/2009 H1N1 pandemic influenza virus (pdmH1N1) were from our laboratory deposits. The A/Aichi/2/1968 H3N2 influenza virus was generously provided by Dr. Dongming Zhou (Tianjin Medical University, Tianjin, China). The SARS-CoV-2 wild-type (WT) virus (CHN/Shanghai_CH-02/2020) and the Omicron XBB variant (CHN/O-XBB-FY.3.1/2023) were maintained in the laboratory of Dr. Ping Zhao (Second Military Medical University, Shanghai, China).

### 2.3 Animals

Female C57BL/6 mice [6-8 weeks old, specific pathogen free (SPF)] were purchased from Shanghai BK Biotechnology Co., Ltd and housed in the animal facility of Shanghai Public Health Clinical Center. Female hACE2-C57BL/6 transgenic mice (6-8 weeks old, SPF) were purchased from Shanghai Model Organisms Center, Inc., and were maintained in the Biosafety Level 3 (BSL-3) laboratory at Second Military Medical University during the study period.

### 2.4 Recombinant vaccinia virus construction

The recombinant vaccinia virus rTTV-RBD-HA2 was developed based on TTV-752-1 by integrating three RBD-HA2 sequences into its genome through homologous recombination. The RBD-HA2 sequences followed the same modality, comprising RBD from SARS-CoV-2, SARS-CoV, or MERS-CoV in fusion with the HA2 extracellular domain of A/Shanghai/02/2013 H7N9 influenza virus, or A/California/04/2009 H1N1 influenza virus, abbreviated as H7 HA2 and H1 HA2, respectively. The SARS-CoV-2 RBD-H7 HA2 and SARS-CoV RBD-H7 HA2 sequences, with a Myc tag and a Flag tag respectively added to the C-terminal for facilitating detection of protein expression, were concatenated using a P2A peptide and inserted into the J2R (thymidine kinase, TK) locus as an expression vector under the control of the early/late viral promoter (PE/L); the MERS-CoV RBD-H1 HA2 sequence was recombined into the A56R locus. The recombinants were selected in 143B cells using BrdU (Roche, Basel, Swiss) and then propagated in DF-1 cells, followed by purification through sucrose density gradient centrifugation. The resultant virus preparations were titrated by plaque assay using 143B cells.

### 2.5 PCR-mediated verification of rTTV-RBD-HA2

PCR analysis was used to confirm that the isolated rTTV-RBD-HA2 candidates have the correct gene modifications. DNA was extracted from DF-1 cells after infection with candidate viruses at a multiplicity of infection (MOI) of 1 for 24 h using Viral Nucleic Acid Extraction Kit II (VR100, Geneaid, New Taipei City, China) following the manufacturer’s instructions. Gene modification events were subsequently examined by PCR analysis using primers located at the TK and A56R gene-flanking regions or primers targeting the inserted gene sequences. The gene insertions were further verified by Sanger sequencing (Tsingke, Beijing, China).

### 2.6 Detection of immunogen expression by rTTV-RBD-HA2 using Western blotting

143B cells were infected with rTTV-RBD-HA2 or TTV-752-1 at a MOI of 1. After 24 h, the cells were harvested and resuspended in RIPA buffer. The resulting lysates were mixed with 4× Protein SDS PAGE Loading Buffer, boiled for 10 min, and then subjected to Western blotting analysis using standard protocols. The primary antibodies used were: a mouse anti Myc-Tag antibody (AE010, ABclonal, Wuhan, China) for detecting the expression of SARS-CoV-2 RBD-H7 HA2, a mouse anti-Flag M2 antibody (F1804, Sigma-Aldrich, Saint Louis, USA) for detecting the expression of SARS-CoV RBD-H7 HA2, a rabbit MERS-CoV Spike antibody (40069-T62, Sino Biological, Beijing, China) for detecting the expression of MERS-CoV RBD-H1 HA2, and a mouse anti-GAPDH antibody (AC002, ABclonal) for detecting GAPDH as the loading control. The secondary antibodies used were an anti-mouse IgG, HRP-linked antibody (7076S, Cell Signaling Technology, Boston, USA) and an anti-rabbit IgG, HRP-linked antibody (7074S, Cell Signaling Technology). Protein bands were visualized by scanning the developed membranes with Odyssey Fc (LI-COR, Lincoln, USA) or ChemiDoc MP (Bio-Rad, Hercules, USA).

### 2.7 Analysis of virus replication

DF-1 cells were infected with rTTV-RBD-HA2 or TTV-752-1 at a MOI of 0.01 for 2 h at 37°C in a 5% CO_2_ incubator. After removing the unabsorbed virus, the cells were washed, and fresh DMEM supplemented with 2% FBS and 1% PS was added before returning the cells to the incubator for further culturing. At 24 h, 48 h, and 72 h post infection, the cells and supernatants were collected and subjected to three freeze-thawing cycles before centrifugation at 3000 g for 10 min. The viral titers of the cleared supernatants were determined by plaque assay in 143B cells.

### 2.8 Immunization and virus challenge of mice

All mouse experiments in this study were approved by the Institutional Animal Care and Use Committee (IACUC) of Shanghai Public Health Clinical Center and Second Military Medical University. Female C57BL/6 mice or female hACE2-C57BL/6 transgenic mice (10 mice per group) were subjected to indicated regimens consisting of either intramuscular immunization with 1×10^7^ PFU of rTTV-RBD-HA2 or TTV-752-1 only, or an intramuscular prime followed by two intranasal boosts, each with a dosage of 3×10^6^ PFU. Sera, splenocytes, and bronchoalveolar lavage fluids (BALF) were collected at indicated time points from vaccinated mice individually to assess humoral and cellular responses. In virus challenge studies, immunized mice were infected with SARS-CoV-2 or influenza viruses using the indicated dose 4 weeks after the last immunization. The subjects were then monitored daily for weight loss and survival for a period of 14 days. Tissues were collected individually 2-3 days post challenge for viral load or viral titer determination and histopathological analysis. For the assessment of the role of lung T cell response in the rTTV-RBD-HA2-mediated protection against pdmH1N1 challenge, immunized mice were divided into three groups: anti-CD8, anti-CD4+CD8, and isotype control. They were respectively given two intranasal injections of 100 µg of an anti-mouse CD8 antibody (BE0061, BioXCell, West Lebanon, USA), 50 µg of the same anti-mouse CD8 antibody combined with 50 µg of an anti-mouse CD4 antibody (C1333, Thermo), or 100 µg of rat IgG2b isotype control (BE0090, BioXCell) on days 4 and 2 before the virus challenge. BAL samples were individually collected from four mice each from the isotype control, anti-CD8, and anti-CD4+CD8 groups on the day of the virus challenge to analyze the presence of CD4+ and CD8+ cells to examine the depletion efficiency. The rest of the mice were challenged with 10 times 50% lethal dose (LD50) of pdmH1N1 virus and subsequently monitored for weight change and survival until 14 days after infection, with a group of mice receiving three intramuscular injections of TTV-752-1 included as the control group. In all virus challenge studies, animals that lost 30% or more of their initial body weight were euthanized and recorded as dead.

### 2.9 Enzyme-linked immunosorbent assay

The vaccine-induced antibodies specific to the three RBDs and the two HAs included in the vaccine, as well as the RBD of the SARS-CoV-2 Omicron XBB variant, were detected using an enzyme-linked immunosorbent assay (ELISA). The 96-well ELISA plates were coated with 1 μg/mL of recombinant SARS-CoV-2 RBD protein (RP01258, ABclonal), SARS-CoV-2 Omicron XBB RBD protein (EVV00331, AntibodySystem, Schiltigheim, France), SARS-CoV RBD protein (RP01299, ABclonal), MERS-CoV RBD protein (RP01311, ABclonal), H7N9 HA protein (40104-V08H, Sino Biological), and H1N1 HA protein (11055-V08B, Sino Biological) at 4°C overnight. After washing the plates with PBST and incubating with blocking buffer (5% skim milk in PBST) for 2 h at room temperature (RT), individual mouse serum or BALF samples were added in 2-fold serial dilutions. After incubating for 3 h at RT, the plates were washed and then incubated with blocking buffer containing HRP-labelled goat anti-mouse IgG (1:5000) (33201ES60, Yeasen, Shanghai, China) or HRP-labelled goat anti-mouse IgA (BF03007, Biodragon, Beijing, China) for 1 h at RT. In the case of differentiating IgG subclass antibodies, the plates were sequentially incubated with biotin-conjugated goat anti-mouse IgG1 (1:5000) or IgG2c (1:5000) (ab97238, ab97253, Abcam, Cambridge, UK) and HRP-streptavidin (1:5000) (35105ES60, Yeasen), with each incubation performed for 1 h at RT. Finally, after extensive washing, the plates were incubated with the substrate OPD (P9187, Sigma-Aldrich), followed by adding 1 M H_2_SO_4_ to stop the reaction, and the absorbance at 490 nm was determined using a Microplate Reader (Cytation 5, BioTek, Vermont, USA). The highest dilution with an absorbance 2-fold higher than the negative control was taken as the endpoint titer.

### 2.10 Enzyme-linked immunospot assay

T cell responses were assessed using a mouse IFN-γ ELISPOT set (551083, BD Biosciences, Franklin Lakes, USA) following the manufacturer’s protocol. In short, the ELISPOT plates were first coated with anti-mouse IFN-γ antibody (5 μg/mL) at 4°C overnight and then blocked for 2 h at RT. Subsequently, 2×10^5^ splenocytes or BAL cells of single mouse were added to each well, followed by stimulation with a peptide pool (Chinapeptides, Shanghai, China) or protein for 20 h at 37°C in a 5% CO_2_ incubator. The plates were then incubated with biotinylated anti-mouse IFN-γ antibody (2 μg/mL) for 2 h at RT. Streptavidin-HRP was subsequently added. After incubating for 1 h at RT, the plates were stained with the AEC substrate reagent and reactions were stopped by adding water until the spots became clearly visible. The developed plates were imaged using an AT-Spot ELISPOT analyzer (Beijing, China); spot-forming cell (SFC) counts were determined using the counting software.

### 2.11 Pseudovirus neutralization assay

Three pseudoviruses carrying the full-length spike protein of the corresponding coronavirus and a luciferase reporter gene were generated for neutralizing assays, following the procedures described in our previous publication (26). In brief, 293T cells were co-transfected with the env-deficient HIV-1 genomic vector pNL4-3.Luc.R-E- (3418, NIH AIDS Reagent Program) and a pcDNA3.1 vector expressing the spike protein (pcDNA3.1-Spike) using EZ Cell Transfection Reagent II (AC04L011, Life-iLab, Shanghai, China). After 48 h incubation at 37°C in a 5% CO_2_ incubator, the pseudovirus-containing supernatants were collected and centrifuged to remove cell debris. The cleared supernatants were filtered through a 0.45 μm filter, aliquoted, and stored at -80°C until use. Each pseudovirus stock was analyzed by a titration assay to determine the dilution used in the neutralization assay. For the neutralizing assay, serial dilutions of heat-inactivated individual mouse serum or BALF were mixed with the appropriately diluted pseudovirus stock. After incubation for 1 h at 37°C, the mixtures were added to pre-plated hACE2-293T cells (SARS-CoV-2 and SARS-CoV pseudoviruses) or Huh-7 cells (MERS-CoV pseudovirus) in 96-well plates. The plates were incubated for 48 h at 37°C in a 5% CO_2_ incubator, and each well was then analyzed for luciferase reporter gene activity using the Bright-Glo Luciferase Assay System (E2650, Promega, Madison, USA) and a luminometer (Promega GloMax 96). The neutralizing titers were defined as the highest dilution achieving a 50% reduction in relative light units (RLUs) relative to virus-only controls after background subtraction.

### 2.12 Quantification of SARS-CoV-2 viral loads by quantitative RT-PCR

The lungs, turbinates, and brains of SARS-CoV-2 challenged hACE2-C57BL/6 transgenic mice were individually collected at indicated time points, pulverized, and solubilized in TRIzol (Thermo). Subsequently, total RNA extraction was performed with a Direct-zol RNA Miniprep Kit (R2052, ZYMO Research, California, USA), followed by reverse transcription using a Reverse Transcription System (A3500, Promega). The resulting cDNAs were then amplified with the GoTaq qPCR Master Mix (A6002, Promega) on a Bioer quantitative real-time PCR system (Hangzhou, China), using the following primer pair specific to the viral N gene: F: 5’-GGGGAACTTCTCCTGCTAGAAT-3’, R: 5’-CAGACATTTTGCTCTCAAGCTG-3’. The thermal program consists of three steps: 95°C for 10 min, 40 cycles of 95°C for 15 s, and 60°C for 1 min.

### 2.13 Determination of lung influenza virus titers

MDCK cells were seeded in 96-well plates and cultured overnight at 37°C in a 5% CO_2_ incubator. Lung tissue homogenate samples from single influenza virus-challenged C57BL/6 mice were obtained as previously described (27) and diluted with DMEM plus 1% PS to create 10-fold serial dilutions. After washing the MDCK cell plates with PBS, 100 μL of the diluted sample was added to each well, followed by incubation for 1 h at 37°C in a 5% CO_2_ incubator. Subsequently, 100 μL of DMEM supplemented with 1% PS and 2 μg/mL TPCK-treated trypsin (T1426, Sigma-Aldrich) was added to each well, and the plates were returned to the incubator for further incubation for 72 h. At the end of the incubation, the culture supernatants were collected, and 25 μL was added to each well of 96-well V-bottom plates along with 25 μL of 1% chicken erythrocytes for incubation for 30 min at RT. Based on the HA assay results, the 50% tissue culture infective dose (TCID50) of each sample was determined using the Reed-Muench method (28).

### 2.14 Histopathology analysis

The lungs from single virus-infected mice were fixed in 4% paraformaldehyde, embedded in paraffin, and cut into sections of 4-5 μm thickness using a microtome. The resulting sections were then subjected to hematoxylin and eosin (H&E) staining and scanned with a TissueFAXS 200 (Tissue Gnostics, Vienna, Austria). The acquired images were analyzed using HistoQuest software. Pathology scores were then calculated based on a published grading system for quantifying lung histopathology associated with respiratory viral infections (29, 30).

### 2.15 Statistical analysis

All statistical analyses were conducted using GraphPad Prism 9 software. Differences between two groups were evaluated using either the t-test or the Mann-Whitney test. A *p*-value less than 0.05 was considered statistically significant.

## 3 Results

### 3.1 Construction and characterization of rTTV-RBD-HA2 vaccine

**Figure 1:**
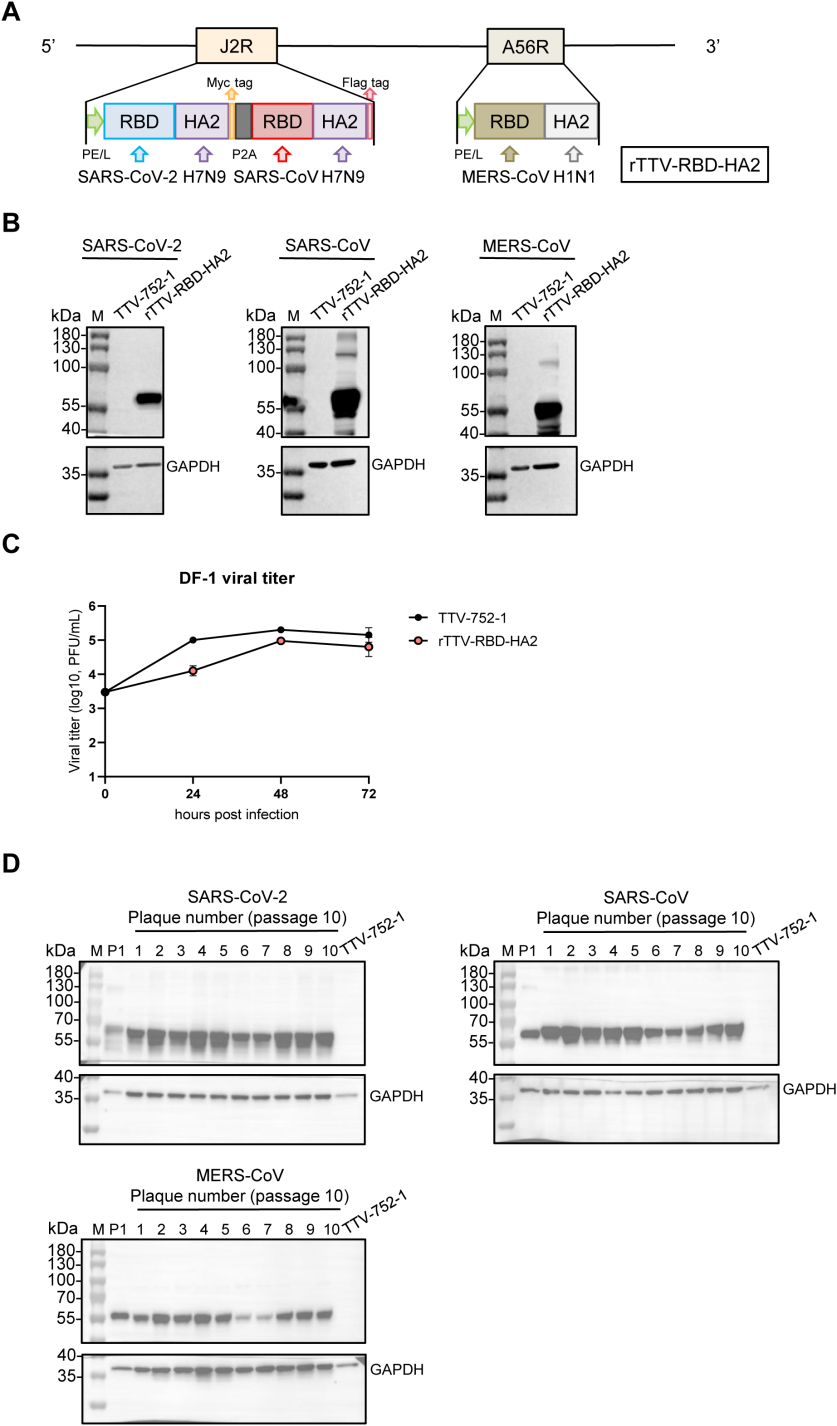
Construction and characterization of the rTTV-RBD-HA2 vaccine. **(A)** Schematic representation of the rTTV-RBD-HA2 vaccine. The vaccine, hereafter referred to as rTTV-RBD-HA2, was engineered by inserting three RBD-HA2 fusion sequences sourced as indicated into the TTV-752-1 vaccinia virus. Two loci were utilized for the insertion: the J2R locus for a tandem of SARS-CoV-2 RBD-H7N9 HA2 and SARS-CoV RBD-H7N9 HA2 linked by the self-cleaving P2A peptide and the A56R locus for MERS-CoV RBD-H1N1 HA2. In both loci, the immunogen expression cassette was under the control of the robust PE/L vaccinia promoter. Additionally, a Myc-tag and a Flag-tag were added C-terminally to the two RBD-HA2 sequences in the J2R locus, respectively, to facilitate detection of their protein expression. **(B)** Immunogen expression of rTTV-RBD-HA2. 143B cells were infected with rTTV-RBD-HA2 or TTV-752-1 at a multiplicity of infection (MOI) of 1 and harvested at 24 h. The resulting whole cell lysates were analyzed by western blotting for the expression of SARS-CoV-2 RBD-H7N9 HA2 (abbreviated as SARS-CoV-2), SARS-CoV RBD-H7N9 HA2 (abbreviated as SARS-CoV) and MERS-CoV RBD-H1N1 HA2 (abbreviated as MERS-CoV), with GAPDH also detected to serve as the loading control. **(C)** Replicative characteristics of rTTV-RBD-HA2 in DF-1 cells. DF-1 cells were infected with rTTV-RBD-HA2 or TTV-752-1 at an MOI of 0.01. Viral titers were measured at 24 h, 48 h, and 72 h post-infection using plaque assays in 143B cells. **(D)** Genetic stability of rTTV-RBD-HA2. Lysates of 143B cells infected for 24 h with the original rTTV-RBD-HA2 stock (P1) or each of the ten individual clones from passage 10 stock were analyzed by western blotting for the expression of the indicated immunogens and GAPDH as the loading control.

In a previous study, we developed a novel immunogen design by fusing the RBD of SARS-CoV-2 with the HA2 of the influenza A virus. With this design, we engineered a single AdC68 (chimpanzee adenovirus 68)-based vaccine and demonstrated its efficacy in protecting mice from the challenges of SARS-CoV-2 and influenza viruses. In this current study, our aim was to further broaden the range of protection offered by such immunogen design. We implemented two key changes to our vaccine construction strategy. Firstly, we transitioned to the Tiantan vaccinia virus strain (TTV-752-1) as the viral vector platform to construct the vaccine. This decision was made because it has a remarkable coding capacity for expressing immunogens, alongside a well-documented safety profile in human use. Secondly, we expanded the immunogen from a single copy of RBD-HA2 to three copies that vary in the source of RBD, covering SARS-CoV-2, SARS-CoV, and MERS-CoV, and the HA2, which is from either pdmH1N1 (A/California/04/2009) or nH7N9 (A/Shanghai/02/2013). Given the higher similarity of SARS-CoV-2 to SARS-CoV compared to MERS-CoV in terms of both genomes and RBD sequences (31), to reduce the risk of recombination between immunogen sequences inserted into different loci, we chose to utilize the J2R (TK) locus for P2A peptide-based co-expressing SARS-CoV-2 RBD-H7 HA2 and SARS-CoV RBD-H7 HA2 under the control of the early/late viral promoter PE/L and the A56R locus to solely express MERS-CoV RBD-H1 HA2, also under the control of PE/L. We named the thus-designed multi-targeting vaccine rTTV-RBD-HA2 (**Figure 1A**).

rTTV-RBD-HA2 was successfully produced through stepwise homologous recombination at the J2R (TK) and A56R loci between the genome of TTV-752-1 and a transfected vector expressing the immunogen expression cassette. We subsequently verified its identity and absence of parental TTV-752-1 contamination through PCR analysis using primers targeting the viral sequence flanking the TK and A56R gene or the immunogen sequences. (**Supplementary Figure 1A**). To evaluate the expression of the immunogens, we infected 143B cells with rTTV-RBD-HA2 at a MOI of 1, using TTV-752-1 as a control. The infected cells were collected at 24 hpi and subjected to Western blotting analysis. As shown in **Figure 1B**, protein bands corresponding to the three RBD-HA2 proteins of the correct size (50-55 kDa) were readily detected in the rTTV-RBD-HA2 sample but not in the TTV-752-1 sample. We further characterized the growth and stability of rTTV-RBD-HA2. When measured in DF-1 cells at an MOI of 0.01, comparing the multiple cycle growth curve of rTTV-RBD-HA2 and the TTV-752-1 parent indicated that rTTV-RBD-HA2 was only moderately attenuated (**Figure 1C**). To assess the stability of rTTV-RBD-HA2, we passaged the virus for 10 consecutive generations and subjected passage 10 to a plaque assay. Ten individual plaques were then picked for DNA extraction and subjected to the same PCR analysis used to verify the initial virus. The results showed that the 10 clones retained all three RBD-HA2 genes and were free of parental TTV-752-1 conversion (**Supplementary Figure 1B**). Western blotting analysis of virus-infected cells further validated the expression of all the immunogens by the 10 virus clones, demonstrating that rTTV-RBD-HA2 remains stable with successive passages (**Figure 1D**).

### 3.2 Systematic and mucosal immunogenicity of rTTV-RBD-HA2 in mice

**Figure 2:**
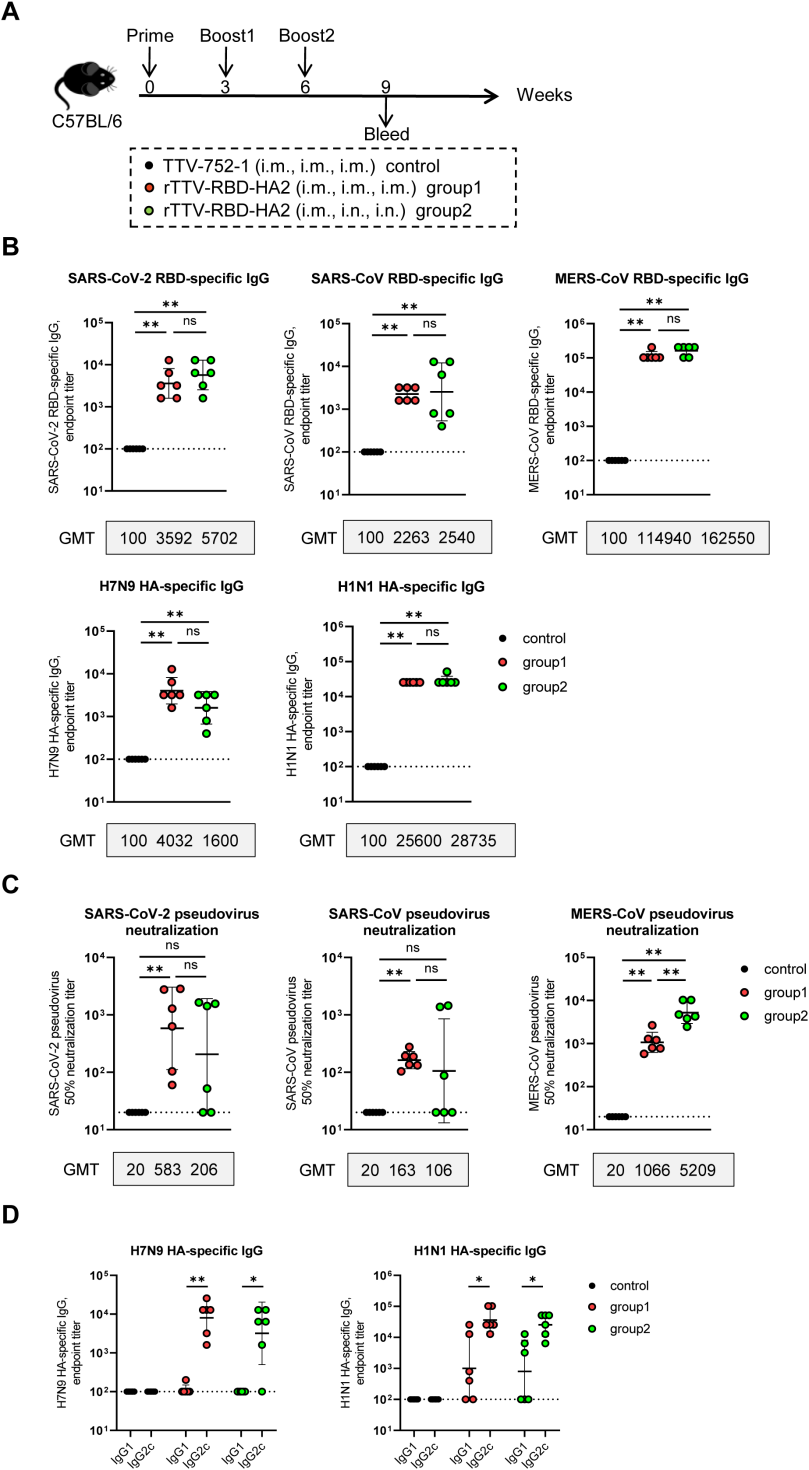
Humoral immune responses of rTTV-RBD-HA2 in C57BL/6 mice. **(A)** Scheme of immunizations and sampling. Female C57BL/6 mice were immunized with 1×10^7^ PFU of rTTV-RBD-HA2 via the intramuscular (i.m.) route at week 0, followed by two booster injections at weeks 3 and 6, each administered through either the i.m. route with a dose of 1×10^7^ PFU (group 1) or the intranasal (i.n.) route with a dose of 3×10^6^ PFU (group 2). Also included was a control group that received three i.m. injections of 1×10^7^ PFU of TTV-752-1 at weeks 0, 3 and 6, respectively. Serum samples were collected at week 9 post the first immunization. n=6 per group. **(B, C)** Assessments of serum samples at week 9 post-prime for RBD- and HA-specific antibody response using ELISA **(B)** and pseudovirus neutralization assay **(C)**. **(D)** ELISA-mediated assessments of IgG2c and IgG1 subtypes, representing Th1 and Th2 responses, respectively, within the serum HA-specific titers. Titer data were presented as geometric mean titer (GMT) ± geometric standard deviation (GSD). The Mann-Whitney test was used to analyze differences between experimental groups. *p<0.05; **p<0.01; ns, no significance.

We next evaluated the immunogenicity elicited by rTTV-RBD-HA2 in C57BL/6 mice under two three-dose regimens consisting of either intramuscular only or an intramuscular prime followed by two intranasal boosts (**Figure 2A**). In the intramuscular immunization-only group (group 1), mice received three intramuscular (i.m.) injections of 1×10^7^ PFU of rTTV-RBD-HA2 at weeks 0, 3, and 6. In the intramuscular prime-intranasal boosting immunization group (group 2), mice were given 1×10^7^ PFU of rTTV-RBD-HA2 via the i.m. route at week 0, followed by two intranasal (i.n.) boosts of a viral dose of 3×10^6^ PFU at weeks 3 and 6. Mice immunized with 1×10^7^ PFU of TTV-752-1 by the i.m. route at weeks 0, 3, and 6 served as the control group.

Serum samples were collected at week 9 post-prime for analysis of target-specific binding antibodies using ELISA and neutralizing antibodies using pseudovirus neutralization assays (n=6 per group). The levels of specific binding antibodies showed no significant difference between the two rTTV-RBD-HA2-vaccinated groups for all five immunogens included in the vaccine. More precisely, the geometric mean titer (GMT) of binding antibodies against SARS-CoV-2 RBD, SARS-CoV RBD, MERS-CoV RBD, and H1N1 HA were higher in group 2 than in group 1. The H7N9 HA-specific antibodies, however, showed the opposite trend, with higher GMT in group 1 compared to group 2 (4032 vs 1600). It should be noted that the levels of MERS-CoV RBD-specific binding antibodies were extremely high for both groups 1 and 2, with a GMT over 110000 (**Figure 2B**). In terms of neutralizing antibody titers against the three targeted coronaviruses, as assessed by pseudovirus neutralization assay, the two vaccination groups showed no significant difference in neutralizing activities against SARS-CoV-2 and SARS-CoV, despite group 1 having higher GMTs than group 2. However, group 2 demonstrated a significant, nearly five-fold higher induction of neutralizing antibodies specific to MERS-CoV compared to group 1 (GMT: 5209 vs 1066, *p*<0.01) (**Figure 2C**). Given the ratio of IgG2c to IgG1 antibodies as a reliable surrogate of the Th1/Th2 immune response (32), we further dissected the HA-specific binding antibodies into IgG2c to IgG1 subtypes by using subtype-specific antibodies in the ELISA. As shown in **Figure 2D**, the results revealed that the average IgG2c/IgG1 ratios for antibodies specific to H7N9 or H1N1 HAs were greater than 1 across the two vaccinated groups, indicating a Th1-biased immune response.

**Figure 3:**
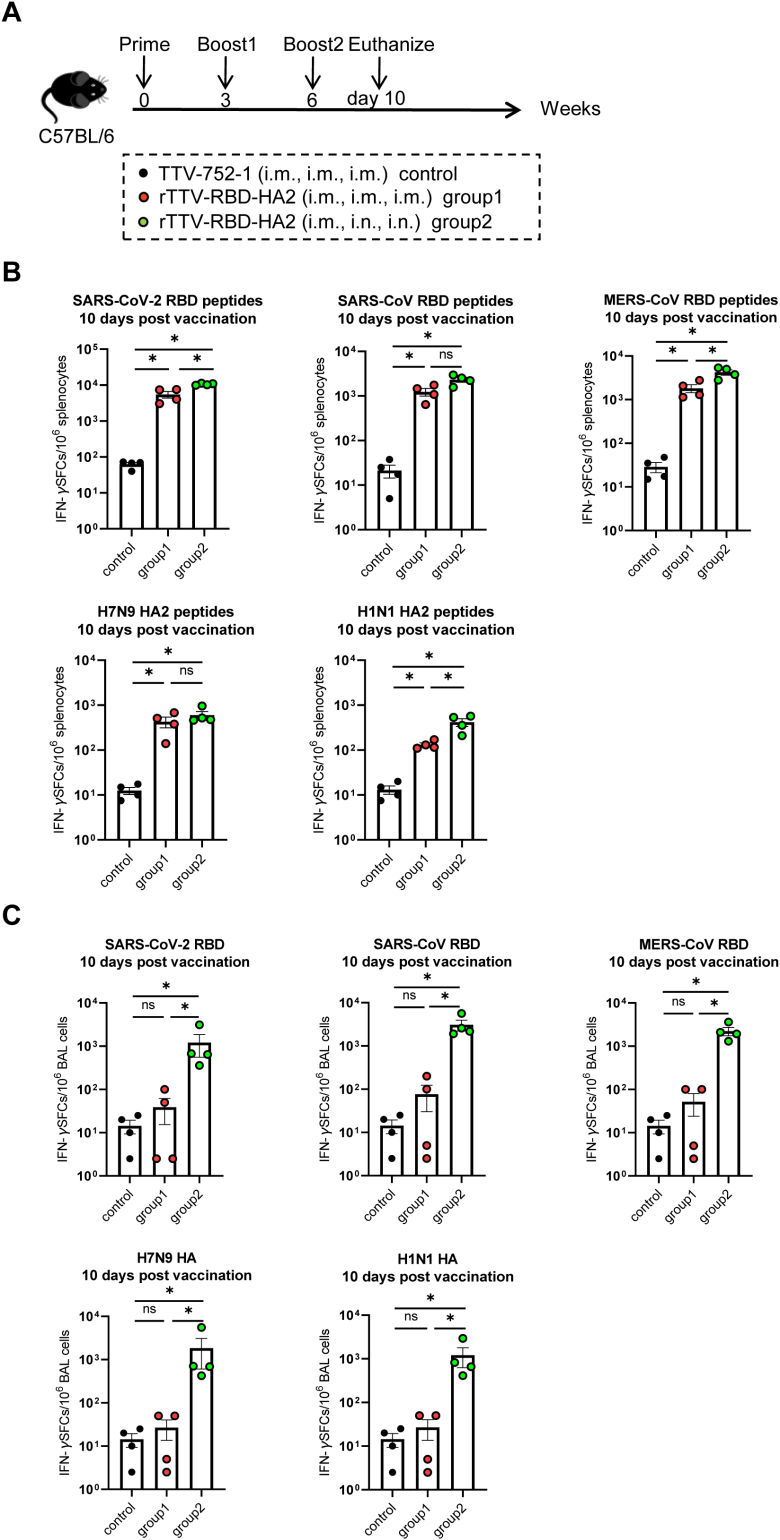
T-cell responses of rTTV-RBD-HA2 in C57BL/6 mice. **(A)** Scheme of immunizations and sampling. Female C57BL/6 mice were given three doses of immunizations with rTTV-RBD-HA2 or TTV-752-1, following the same regimens and animal grouping as outlined in **Figure 2A**. Animals were euthanized on day 10 after the final immunization to sample spleens and bronchoalveolar lavage (BAL) cells. n=4 per group. **(B, C)** Assessment of T-cell responses. Splenocytes **(B)** and BAL cells **(C)** sampled on day 10 after the final immunization were stimulated with the indicated peptide pools and proteins, respectively. The number of IFN-γ-secreting cells was then determined by ELISPOT. ELISPOT data were presented as mean ± standard error of the mean (SEM). The Mann-Whitney test was used to analyze differences between experimental groups. *p<0.05; ns, no significance.

We also conducted analyses of the vaccine-induced cellular immune responses at 10 days post final vaccination (**Figure 3A**) (n=4 per group). The systemic and respiratory T-cell responses were evaluated by stimulating splenocytes and bronchoalveolar lavage (BAL) cells from vaccinated animals with the RBD peptide pools of the targeted three coronaviruses, as well as the HA2 peptide pools of H7N9 and H1N1, and RBD and HA proteins, respectively. The levels of IFN-γ-secreting cells thus induced were then determined using enzyme linked immunospot (ELISPOT) assay. As shown in **Figure 3B**, in response to stimulation with each of the three RBD peptide pools and the two HA2 peptide pools, splenocytes from both rTTV-RBD-HA2-vaccinated groups exhibited significantly higher levels of IFN-γ-secreting cells, quantified as spot-forming cells (SFC) per 1×10^6^ splenocytes, compared to the control group. Between the two rTTV-RBD-HA2-vaccinated groups, group 2 showed a more robust overall T-cell response, with a significantly higher induction of IFN-γ-secreting cells observed for three out of the five peptide pools (SARS-CoV-2 RBD, MERS-CoV RBD, and H1N1 HA). Compared to splenocytes, the analysis of BAL cells revealed a much more marked difference between the two rTTV-RBD-HA2-vaccinated groups. BAL cells from group 2 remained reactive to the stimulation of all five proteins, with an average count of SFCs per 10^6^ BAL cells ranging from 1200 to 3000. In contrast, BAL cells from group 1 showed significantly much lower induction of T cell response to all the stimulations, only slightly higher than the background levels observed with the control group (**Figure 3C**). Meanwhile, we also evaluated the humoral responses in the BALF. Surprisingly, we failed to detect immunogen-specific IgA antibodies in all three groups (**Supplementary Figure 2B**). Consistent with this, neither of the rTTV-RBD-HA2-vaccinated groups showed effective induction of coronavirus-neutralizing titers in BALF. Among the three neutralizing activities measured, the MERS-CoV neutralizing activity was the only one showing a significant difference between the rTTV-RBD-HA2-vaccinated and control group. It reached the highest GMT of all measured at 53 in group 2 samples, which was more than 5-fold higher than in group 1 samples (**Supplementary Figure 2C**). These assessments together demonstrated that the rTTV-RBD-HA2 vaccine was capable of eliciting potent systemic humoral and cellular immunity against the three coronaviruses and the two influenza viruses it intended to target through an intramuscular only or intramuscular prime-intranasal boosting regimen. However, only the latter allowed for the effective induction of mucosal immunity, primarily consisting of a balanced, broad T-cell response.

To determine if rTTV-RBD-HA2 has the optimal RBD-HA2 arrangement, we compared its immunogenicity to an alternative version where the SARS-CoV RBD was paired with H1N1 HA2 while keeping all other elements and insertion sites the same. This comparison was conducted using an i.m. prime-i.m. boost regimen, and the induced immune response was analyzed at week 7 after the initial immunization (**Supplementary Figure 3**). We observed that the alternative version failed to raise H7N9 HA-specific antibodies as rTTV-RBD-HA2 did (**Supplementary Figure 3B**). One plausible explanation is that the intrinsic immunogenicity of H7N9 HA2 is weaker than that of H1N1 HA2, thus requiring two copies in the vaccine for an effective induction of its specific immune response. This result demonstrates that rTTV-RBD-HA2 has an optimal RBD-HA2 arrangement.

### 3.3 Protection against SARS-CoV-2 challenge by rTTV-RBD-HA2 vaccine in hACE2-C57BL/6 transgenic mice

**Figure 4:**
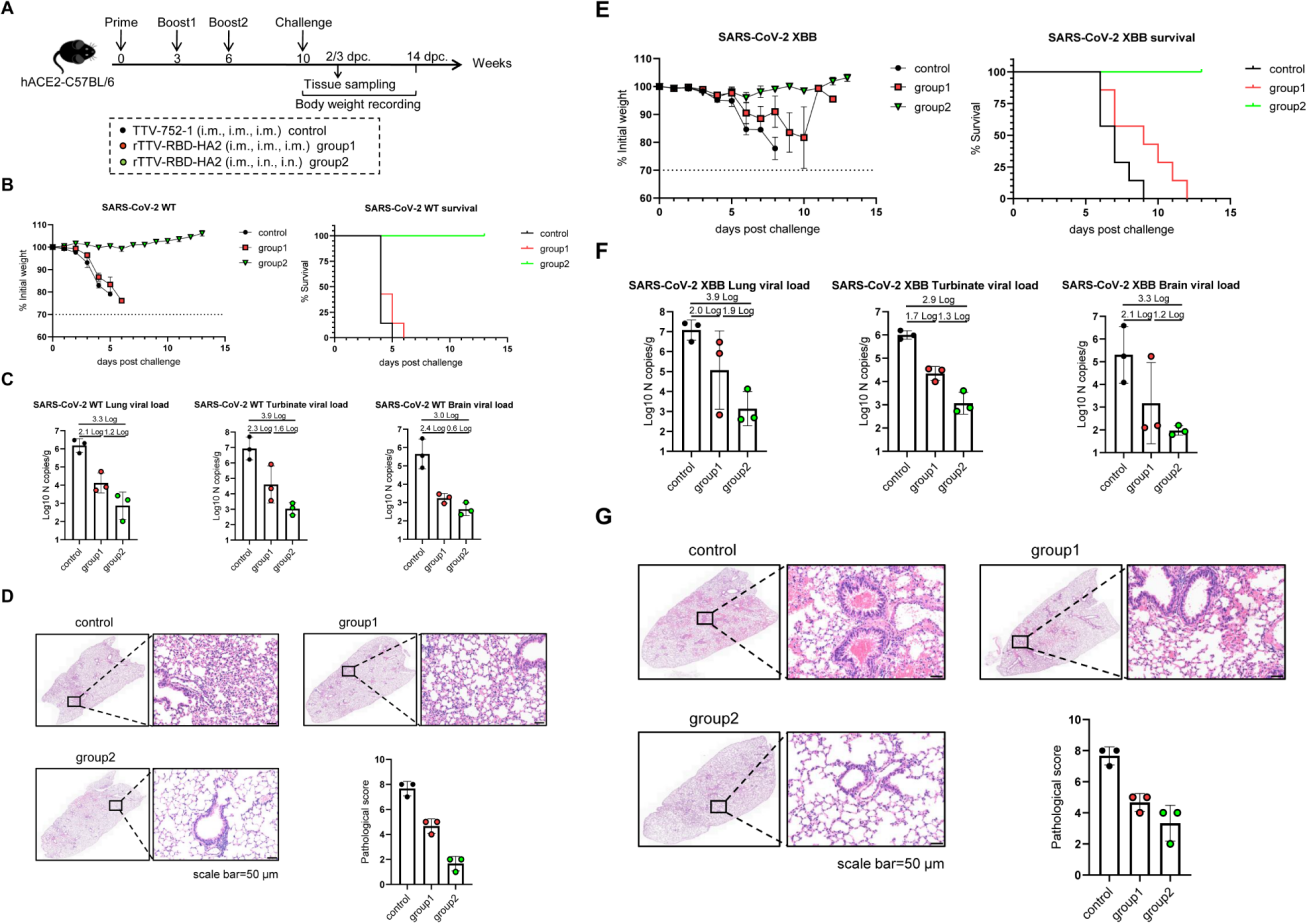
Protective efficacy of rTTV-RBD-HA2 against SARS-CoV-2 challenge in hACE2-C57BL/6 transgenic mice. **(A)** Scheme of experimental schedule. Female hACE2-C57BL/6 transgenic mice were divided into three groups and vaccinated with a three-dose, nine-week regimen of either TTV-752-1 (control) or rTTV-RBD-HA2 (group 1: i.m. alone; group 2: i.m.+i.n.), with administration dosage and timing the same as outlined in **Figures 2A** and **3A**. All the mice were challenged with 1000 PFU of SARS-CoV-2 WT or SARS-CoV-2 XBB via i.n. route at week 10 post the first immunization. A portion of each group (n=3) was sacrificed at the indicated time points to collect tissues for viral load determination and histopathological analysis. The remaining animals were monitored for 14 days to track survival and weight changes. n=7 per group. **(B-D)** Assessments of rTTV-RBD-HA2-mediated protection against the SARS-CoV-2 WT challenge. In **(B)**, body weight curves (left) and survival curves (right) are displayed over a 14-day observation period. In **(C)**, viral loads in the lungs, turbinates, and brains collected on day 2 after the viral challenge are shown. These were measured using quantitative RT-PCR analysis of viral N transcripts and expressed as log10 copies per gram (tissue weight). In **(D)**, representative images of H&E-stained sections of lungs collected on day 2 after the infection are shown (scale bar, 50 μm) alongside quantified pathological scores (expressed as mean ± SD). **(E-G)** Assessments of rTTV-RBD-HA2-mediated protection against the SARS-CoV-2 XBB challenge, including survival and weight change data **(E)**, tissue viral load data **(F)**, and representative stained lung sections and quantified pathological scores **(G)**. The assessments followed the same outline described in **(B-D)** for the SARS-CoV-2 XBB challenge, with the only difference being that tissue collections were performed on day 3 instead of day 2 after the challenge.

We then used hACE2-C57BL/6 transgenic mice to evaluate the protective efficacy of the rTTV-RBD-HA2 vaccine against SARS-CoV-2 infection. To assess the extent of this protection, we challenged the immunized mice with wild-type (WT) SARS-CoV-2 as well as the Omicron XBB variant. The mice were divided into three groups: two experimental groups, groups 1 and 2, were immunized with three doses of rTTV-RBD-HA2 using either an intramuscular-only regimen or an intramuscular prime-intranasal boosting regimen; mice receiving three doses of TTV-752-1 via the same intramuscular-only regimen served as the control group. The conditions of the two immunization regimens were the same as described above. The animals were analyzed at week 9 post-prime for serum antibody response and challenged with WT SARS-CoV-2 and the Omicron XBB variant a week later. The infected mice were monitored for up to 14 days for survival and weight changes, with some sacrificed at 2 days post challenge (dpc) for WT SARS-CoV-2 infection or 3 dpc for the Omicron XBB variant infection to collect tissues for viral load determination and histopathological examination (**Figure 4A**).

As analyzed in the week 9 post-prime, similar serum binding antibody titers against SARS-CoV-2 WT RBD were readily detected in the two experimental groups (GMT: 20794 vs 23886). The two groups also exhibited comparable titers of SARS-CoV-2 XBB RBD-specific binding antibodies. However, the titers (GMT: 2425 vs 2986, **Supplementary Figure 4A**) were significantly lower than those against SARS-CoV-2 WT RBD. This result was expected as it is consistent with the nature of XBB as a highly effective antibody-evading variant. There was no significant difference between the two experimental groups in serum SARS-CoV-2 WT-specific neutralizing activity, although group 1 exhibited a higher GMT than group 2 (316 vs 222). In contrast, we could not detect neutralizing antibodies against SARS-CoV-2 XBB above background levels for both groups (**Supplementary Figure 4B**). We further analyzed the partition of IgG1- and IgG2c-subtype binding antibodies against SARS-CoV-2 WT and SARS-CoV-2 XBB. The results showed that for both binding antibodies, the IgG2c titer was higher than the IgG1 titer, consistent with our conclusion that the antibody response to the rTTV-RBD-HA2 vaccine is likely Th1-biased (**Supplementary Figure 4C**).

The subsequent virus challenge studies revealed marked differences between the three groups. Following the SARS-CoV-2 WT challenge, most of the mice in the control group died on 4 dpc and all succumbed on 5 dpc. All mice in group 1 died on day 6. In contrast, group 2 exhibited a 100% survival rate during the 14-day observation period. Consistent with the difference in survival, unlike the rapid weight loss seen in the control group and group 1, the body weights of mice in group 2 remained stable throughout the observation period (**Figure 4B**). We assessed the viral loads in the lung, turbinate, and brain on 2 dpc by qPCR measurement of the viral N transcripts. High viral loads were detected in all three tissues in the control group, with the turbinate having the highest level. Compared to the control group, group 1 showed a 2.1-log, 2.3-log, and 2.4-log reduction in lung, turbinate, and brain viral loads, respectively. These numbers were further increased to 3.3-log, 3.9-log, and 3-log in group 2, indicating heightened viral inhibition (**Figure 4C**). The superior protection of group 2 was also supported by histopathological analysis of the lungs. Group 2 showed more improved lung pathology than group 1 relative to the control, marked by a significantly greater reduction in inflammatory infiltrates (**Figure 4D**). Compared to the WT SARS-CoV-2 challenge, the Omicron XBB challenge was characterized by a delayed onset of body weight loss beginning on 6 dpc. After this point, mice in the control group experienced a significant weight decline, leading to complete mortality by 9 dpc. In stark contrast, mice in group 2 showed little effect of the viral challenge on body weights and were all alive at the end of the 14-day observation period. Group 1 exhibited moderate improvements in weight loss compared to the control group, but still had similar weight loss dynamics. Consequently, all members succumbed by 12 dpc (**Figure 4E**). The viral load measurements and histopathological examinations further revealed a better protective effect in group 2. Group 2 exhibited a greater degree of reduction in viral loads compared to the control group than group 1 in all three examined tissues: 3.9-log vs. 2.0-log in the lung, 2.9-log vs. 1.7-log in the turbinate, and 3.3 log vs. 2.1 log in the brain (**Figure 4F**). They also demonstrated a significant improvement in lung histopathology, characterized by a notably reduced hemorrhage (**Figure 4G**). Thus, rTTV-RBD-HA2 could offer effective protection against the SARS-CoV-2 WT and the Omicron XBB variant when administered through the intramuscular prime-intranasal boosting regimen. Conversely, the intramuscular-only regimen enabled only partial protection at best, suggesting the essential involvement of a mucosal immune response.

### 3.4 Protection against pdmH1N1 and H3N2 challenge by rTTV-RBD-HA2 vaccine in C57BL/6 mice

**Figure 5:**
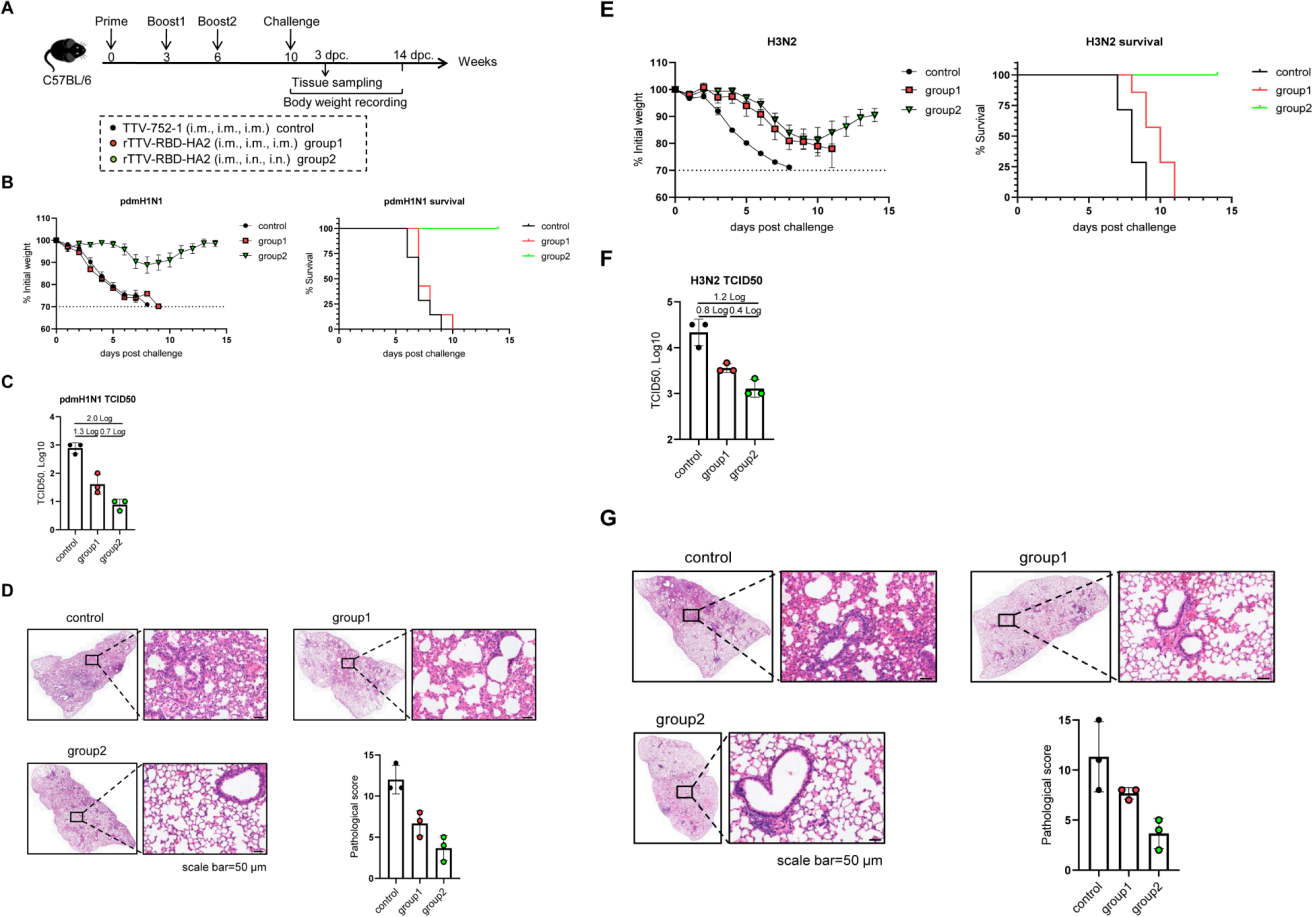
Protective efficacy of rTTV-RBD-HA2 against influenza pdmH1N1 and H3N2 virus challenges in C57BL/6 mice. The animal grouping and immunizations with rTTV-RBD-HA2 or TTV-752-1 followed the protocol shown in **(A)**, which was identical to that shown in **Figure 4A**, except that female C57BL/6 mice (n=10) were used. Ten weeks after the initial immunization, mice were intranasally exposed to 10 times LD50 of influenza pdmH1N1 or H3N2 viruses. The protective efficacy was assessed using three measurements: body weight and survival over a 14-day observation period **(B, E)**, lung viral titers on day 3 post virus challenge, determined as log10 TCID50 titer per gram using MDCK cells **(C, F)**, and histopathological analysis of lungs on day 3 post virus challenge, with representative H&E stained sections alongside quantified pathological scores shown in **(D, G)** (scale bar, 50 μm).

We also assessed the efficacy and breadth of the influenza-protective arm of the rTTV-RBD-HA2. The grouping and immunization of mice with either rTTV-RBD-HA2 or TTV-752-1 were the same as in the SARS-CoV-2 challenge studies. Virus challenges were carried out at week 10 post-prime with i.n. administration of 10 times LD50 of pdmH1N1 or H3N2, representing group 1 and group 2 influenza viruses, respectively. Following infection, the mice were monitored for weight changes and survival for 14 days, and lungs were harvested on 3 dpc for assessing viral titers and histopathology (**Figure 5A**).

After the pdmH1N1 challenge, the mice in the control group and group 1 continued to lose weight and all died by 9 dpc and 10 dpc, respectively. In contrast, the body weight of mice in group 2 was relatively stable until 6 dpc, when a moderate loss began. However, they started to gain weight from 8 dpc and all survived at the end of the observation period (**Figure 5B**). The lung viral titer measurements and histopathological analyses were consistent with the body weight and survival data. In comparison to the control group, the lung viral titers were reduced to a greater extent in group 2 than in group 1 (a 2-log reduction vs a 1.3-log reduction) (**Figure 5C**). Lungs from group 2 also exhibited improved histopathology, with significantly fewer infiltrating inflammatory cells, than those from group 1 (**Figure 5D**). We also observed a protective advantage of group 2 over group 1 upon the H3N2 challenge. Despite experiencing similar weight loss dynamics, the mice in group 2 showed signs of recovery starting on 11 dpc. A more pronounced difference was observed in survival rates: the control group and group 1 completely succumbed by 9 dpc and 11 dpc, respectively, while all mice in group 2 survived at the end of the observation period (**Figure 5E**). In terms of lung viral titers, group 2 showed a 1.2-log reduction compared to the control group, which was higher than the 0.8-log reduction exhibited by group 1 (**Figure 5F**). The better protection of mice in group 2 than group 1 was also reflected in a more reduced lung pathology (**Figure 5G**). These results collectively validated the capability of the rTTV-RBD-HA2 to confer cross-group protection against influenza viruses. They also further emphasized the importance of combining intramuscular and intranasal routes in the immunization regimen to maximize the protection efficacy.

### 3.5 Importance of lung-resident T cells in rTTV-RBD-HA2-mediated protection against pdmH1N1 challenge

**Figure 6:**
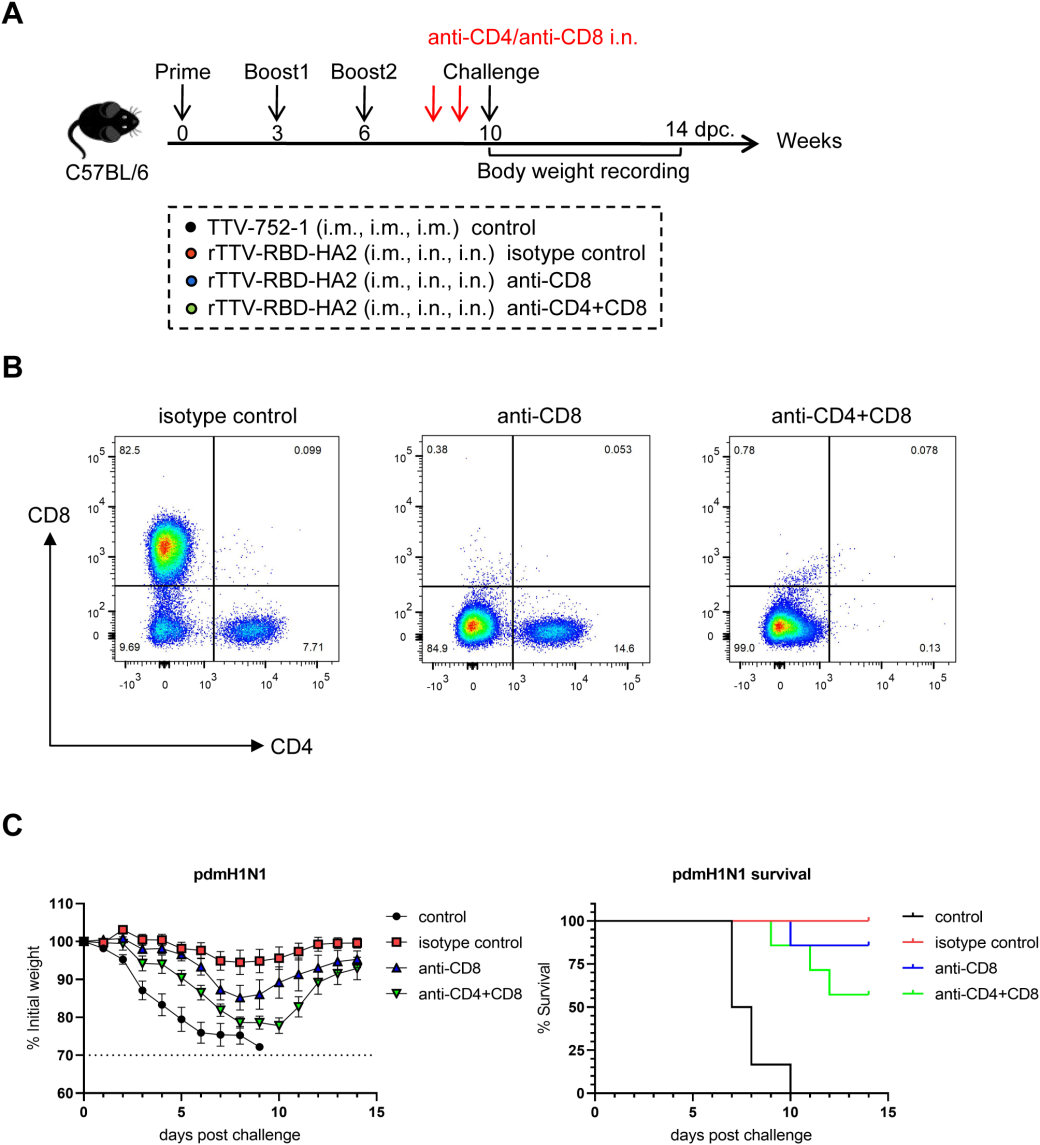
Assessment of the role of lung-resident T cells in rTTV-RBD-HA2-mediated protection against pdmH1N1 challenge by antibody-mediated depletion. **(A)** Experimental schedule and animal grouping information. Depletion of T cells in the lungs was achieved by intranasal administration of an anti-CD8 antibody (the anti-CD8 group), a combination of the same anti-CD8 antibody and an anti-CD4 antibody (the anti-CD4+CD8 group), and an isotype control antibody (the isotype control group) on days 4 and 2 before virus challenge. n=11 per experimental group. **(B)** Assessment of efficiency of T cell depletion in the lung by flow cytometry. Four mice from the three antibody-treated groups were sacrificed on the day of the virus challenge, BAL cells were then collected for analyzing the presence of CD4+ and CD8+ T cells using flow cytometry. **(C)** Comparison of weight change curve and survival curve among the four experimental groups during a 14-day observation period (n=7).

Finally, we sought to determine the roles of lung-resident T cells in rTTV-RBD-HA2-mediated protection. To this end, we performed immunization and pdmH1N1 challenge of mice following the same schedule as depicted in **Figure 5**, with the addition of an injection of an anti-mouse CD8 antibody (anti-CD8), a combination of the same anti-mouse CD8 antibody and an anti-mouse CD4 antibody (anti-CD4+CD8), or isotype-matched antibodies (isotype control) 4 and 2 days before the challenge. Also included were the TTV-752-1-immunized mice as the control group (control) (**Figure 6A**). On the day of the virus challenge, BAL cells were individually collected from four mice from the anti-CD8 group, anti-CD4+CD8 group and the isotype control group and analyzed for the presence of CD4+ and CD8+ T cell subsets using flow cytometry. The analysis results demonstrated that CD4+ and CD8+ T-cell subsets were almost completely depleted in the BAL sample from the anti-CD4+CD8 group compared to the isotype control group, validating the effectiveness of the anti-CD4 and anti-CD8 antibodies used (**Figure 6B**). The rest of the animals were challenged with 10 times LD50 of pdmH1N1 virus and subsequently observed for weight loss and survival for 14 days. Consistent with our previous challenge studies with the pdmH1N1 virus, the entire control group died within 10 days after virus infection, while the vaccinated and isotype antibody-treated group all survived by the end of the observation period. Double depletion of CD4+ and CD8+ cells was associated with marked weight loss in the early phase after infection, though to a lesser extent than the control group and exhibiting a later recovery, alongside a reduction of the survival rate to approximately 60%. Interestingly, depleting only CD8+ T cells also decreased protectivity, as judged by both weight loss and survival rate, but the effect was less pronounced than the double depletion (**Figure 6C**). Collectively, these results establish that the lung-residing T cells, potentially both CD4+ and CD8+ T cells, are involved in but not solely responsible for the influenza-targeting protection afforded by rTTV-RBD-HA2 through the systemic priming-mucosal boosting regimen.

## 4 Discussion

The continuing evolution of SARS-CoV-2 and the associated enhanced immunity-evading capability elaborates its identity as a long-standing human health threat, promoting further development of more effective vaccines with broad-spectrum protectivity. Before SARS-CoV-2, the influenza A virus was the predominant RNA virus responsible for respiratory infections and diseases. It posed a daunting challenge for vaccination-mediated protection, as the efficacy of the inactivated vaccines was consistently subpar and varied from season to season. This was due to the virus’s wide genetic diversity, which is further enhanced by a high mutation rate and within-cell recombination, leading to antigenic drift and shift, respectively. Thus, the vaccination program against respiratory viral infections should consider SARS-CoV-2 and the influenza virus. It is crucial to recognize that other coronaviruses of zoonotic origin have caused epidemics in the past with higher mortality rates than SARS-CoV-2, such as SARS-CoV-1 and the still circulating MERS-CoV. An optimal vaccine should also provide coverage for these viruses. Along this line, several studies have explored vaccine strategies to simultaneously prevent SARS-CoV-2 and influenza virus infections. These strategies include the combination of two mRNA vaccines encoding HA and RBD respectively (33), an adenovirus-based vaccine co-expressing chimeric RBDs and HA (34), co-formulated two-in-one inactivated vaccines against SARS-CoV-2 and influenza viruses (35), an RBD-incorporated hybrid influenza virus-like particle (VLP) vaccine (36), and attenuated or replication-defective influenza virus engineered to express RBD protein from its genome (37). When tested in animal models, these vaccines showed promise in affording dual protection against SARS-CoV-2 and influenza viruses, with the coverage of the former ranging from wild-type only to pan-variants and that of the latter primarily restricted to the subtype corresponding to the HA encoded in the vaccine. In this study, we expanded this coverage to a higher level by engineering a vaccinia-based vaccine, rTTV-RBD-HA2, based on an RBD-HA2 modality we previously designed and demonstrated its dual targeting capacity in mouse infection models (24). Constructed by incorporating three RBD-HA2 moieties pairing RBD from SARS-CoV-2, SARS-CoV, or MERS-CoV with HA2 from nH7N9 or pdmH1N1, rTTV-RBD-HA2 was able to induce a Th1-biased systemic immune response with a multi-targeting profile that includes efficient elicitation of neutralizing antibodies against the three targeted coronaviruses, antibodies reactive to the two influenza virus targets, and T-cell responses to all five encoded viral immunogens, in mice. We demonstrated this induction with a three-dose immunization, which could be administered intramuscularly alone or with intramuscular priming followed by intranasal boosting. However, only the latter regimen was able to promote the formation of mucosal immunity in the lung. This immunity was characterized by a potent T cell response against the RBD and HA, with less effective antibody induction. We further explored the protective efficiency of the two regimens in mouse infection models. The intramuscular priming-intranasal boosting regimen once again demonstrated superiority over the intramuscular alone regimen, showing more pronounced protection against both challenges of SARS-CoV-2 and influenza viruses. Importantly, this protection was not only effective but also broad, covering SARS-CoV-2 pandemic strains including WT and the Omicron XBB subvariant, along with cross-group coverage of influenza viruses, as demonstrated with the pdmH1N1 and H3N2 strains. Finally, through antibody-mediated depletion, we were able to demonstrate that lung-resident T cells play a significant role in the protection afforded by rTTV-RBD-HA2 against pdmH1N1. Interestingly, such contribution appears to involve both CD4+ and CD8+ T cell subsets.

Our results validated the effectiveness of the RBD-HA2 fusion as an immunogen designed to engage protective antibody response targeting RBD and HA2. Importantly, the rationale for this design is supported by a recent structural study showing that an RBD-HA chimeric protein, consisting of the RBD from the SARS-CoV-2 delta variant and the HA stalk from an H1N1 influenza virus, forms a stable trimer in solution while maintaining accessibility to both RBD- and HA-targeting antibodies (38). Our analysis of rTTV-RBD-HA2 also confirmed the suitability of using H1N1 HA2 to construct the RBD-HA2. Its combination with the MERS-CoV RBD allowed for potent induction of MERS-CoV neutralizing antibodies with titers higher than the induced neutralizing antibodies specific for SARS-CoV and SARS-CoV-2 arising from the two other RBD-HA2 immunogens, both adopting H7N9 HA2. This advantage, albeit to a lesser extent, was also reflected in the HA-specific antibody titers. Thus, the broad-spectrum coverage for both coronaviruses and influenza viruses by rTTV-RBD-HA2 lies in the plasticity of the RBD-HA2 design in accommodating combinations of different RBDs and HA2s. The successful generation of rTTV-RBD-HA2 also benefits from the capability of the vaccinia vector to tolerate the insertion of large-size foreign DNA. Our primary reason for utilizing two loci to integrate the three immunogens was to prevent potential homologous recombination among the immunogens, particularly for the two sharing the H7 HA2 sequence. Indeed, we confirmed the proper expression of the three encoded immunogens by rTTV-RBD-HA2 and further showed that it is stable for at least ten generations after propagation. Disrupting two loci might introduce the risk of virus attenuation compared to a single locus, which could affect the immunogenicity of the resulting vaccine. We demonstrate that this concern did not apply to our design, as rTTV-RBD-HA2 only showed moderate attenuation compared to the parental TTV-752-1.

The route of vaccination is a crucial factor in determining the efficacy of a given vaccine by influencing the localization of the induced immune response (39, 40). Testing different vaccination routes is particularly important for a vaccine against respiratory infection, as an effective induction of mucosal immunity has consistently emerged as a correlate of protection alongside the systemic immune response. Aligning with this is the conclusion from previous studies on various types of SARS-CoV-2 vaccines that vaccination through an intramuscular-only regimen is limited in directing effective immunity to the respiratory mucosa (41, 42). For viral vector-based vaccines, utilizing a mucosal boost has an additional benefit in reducing the vector effect imposed by pre-existing antibodies against the vector, thus enabling more effective boosting. Before this study, several groups had used viral vectors, including modified vaccinia virus Ankara (MVA), as backbones to create SARS-CoV-2 vaccines. They collectively demonstrated that intranasal inoculation was essential for these vaccines to drive protective immunity in the lungs (34, 43, 44). Consistent with these studies, our characterizations of rTTV-RBD-HA2 in a three-dose regimen also highlighted the importance of incorporating intranasal boosting. Specifically, we found that out of a schedule consisting of only intramuscular administration and a schedule combining an intramuscular prime and two intranasal boosts, only the latter was able to induce potent T-cell responses in the lungs against all five viral targets that rTTV-RBD-HA2 is designed for. In contrast, both schedules were able to elicit a balanced and robust systemic immunity, involving both neutralizing antibodies and antigen-specific T cells. Although we did not delve into the individual contributions of intramuscular and intranasal routes in the prime-boost regimen, we envision that the regimen likely follows the prime-pull strategy as previously demonstrated with various mucosa-targeting vaccines (45–47). In this strategy, the pre-existing systemic immunity established by the parenteral route can be leveraged by the subsequent mucosal route to enhance the effective induction of mucosal immunity. Interestingly, despite a strong systemic antibody response, we failed to detect a significant induction of antibody responses in the lungs of the animals receiving the intramuscular prime-intranasal boosts, except for low levels of neutralizing antibodies against MERS-CoV. Thus, the mucosal immunity achieved by rTTV-RBD-HA2 in the study is heavily centered on T-cell response.

Our comparison of the two vaccination schedules in protecting immunized mice against SARS-CoV-2 and influenza challenges also revealed a marked advantage of the intramuscular prime-intranasal boost over the intramuscular-only schedule. When testing protection against SARS-CoV-2 on WT and the XBB omicron subvariant using hACE2-C57BL/6 transgenic mice, the intramuscular prime-intranasal boost conferred full protection against both pandemic strains, evidenced by complete survival, no weight loss, and potent suppression of viral loads in the lung, turbinate, and brain. In contrast, animals that received the intramuscular-only schedule all died within the 14-day observation period, with protection only shown in slightly alleviated weight loss and delayed death in the case of the XBB challenge. This inferior protection was consistent with significantly less control of viral loads in all three tissues examined and aggravated lung pathological changes. Our evaluations of the influenza-protecting efficacy of rTTV-RBD-HA2 were conducted using C57BL/6 mice against one group 1 virus, the pandemic pdmH1N1 strain, and one group 2 virus, an H3N2 strain. While the intramuscular prime-intranasal boost schedule enabled full protection against both viruses, the only effect we observed with the intramuscular-only schedule was improved weight loss and delayed animal death in the H3N2 challenge. It should be noted that the RBD sequence employed in the rTTV-RBD-HA2 was from the WT strain while the HA2 sequence was from the H7N9 strain. Thus, the observed complete protection against the Omicron XBB subvariant and the heterologous H3N2 challenges strengthens our conclusion that rTTV-RBD-HA2 met our expectation for its design as a mucosal vaccine simultaneously targeting severe coronaviruses and influenza A viruses with a cross-group coverage.

Coupling immune response profiling with protectivity data lends insight into the immune mechanisms governing rTTV-RBD-HA2-mediated protection. The main difference we identified between the two regimens explored in the study is that only the intramuscular prime-intranasal boosts could establish a potent lung T-cell response. On the other hand, the two regimens were characterized by similar engagement of systemic B and T cell responses. Thus, the most reasonable explanation for only intramuscular prime-intranasal boosts being able to confer effective protection against SARS-CoV-2 and influenza viruses is that a systemic B and T cell response is insufficient for protection, requiring the involvement of lung T cell immunity. Our T cell depletion experiment confirmed this explanation by demonstrating that removing lung T cell response significantly reduces the effectiveness of rTTV-RBD-HA2 achieved through the intramuscular priming-intranasal boosting regimen. Interestingly, we observed less of an effect from depleting CD8+ T cells compared to the double depletion of CD4+ and CD8+ T cells, indicating the involvement of both T cell subsets. This is consistent with previous studies on various types of influenza vaccines showing that CD4+ T cells act alongside CD8+ T cells to contribute to the vaccine’s effectiveness (48, 49). It should also be noted that, even without lung T cell response, rTTV-RBD-HA2-vaccinated mice still exhibited partial protection against pdmH1N1 challenge. This remaining protection is likely provided by circulated virus-specific T cells, which show more effective induction by the intramuscular priming-intranasal boosting regimen compared to the intramuscular alone regimen. These cells should largely be immune from the localized action of nasally administered depleting antibodies.

Our results align with a growing body of evidence supporting that potent T cell immunity, particularly the elicitation of lung-resident T cells, is key to effective defense against respiratory viral infections. One study discovered that the total number of lung-resident T cells correlated with protection against severe COVID-19 (50). We also learned a valuable lesson from our previous exploration of a T cell-based universal AdC68 influenza vaccine, where activating both respiratory resident memory T cells and systemic memory T cells was required to maximize the protection (51). It is plausible to speculate that rTTV-RBD-HA2-mediated protection through the intramuscular prime-intranasal boosts receives a tripartite contribution from systemic B cells and the produced neutralizing antibodies, systemic memory T cells, and lung resident T cells. A tantalizing emerging notion is that T cell immunity may act independently of the antibody response to defend effectively against respiratory infections, as initially suggested by the presence of highly SARS-CoV-2-exposed seronegative individuals (52–54). This notion has received further support from clinical observations of individuals with B cell deficiency that links an enhancement of T cell responses to SARS-CoV-2 infection and vaccination to a reduced risk of developing severe COVID-19 (55). Based on the IgG antibody subtype analyses, rTTV-RBD-HA2 induced a Th1-biased immune response in mice when administered through both vaccination regimens. IFN-γ, the most prominent cytokine secreted by Th1 cells, is a critical tool of T cells used to antagonize viral infection (56). Its prompt production by lung tissue-resident CD8+ T cells in response to viral invasion and subsequent action in driving a tissue-wide interferon-induced genes (ISGs) induction has been identified as a frontline protective mechanism against respiratory viral infection (57). Several studies have shown cross-reactivity of T cell responses to different SARS-CoV-2 strains (58), which might account for rTTV-RBD-HA2 maintaining a protective effect against the Omicron XBB subvariant despite only containing WT RBD as the immunogen. Cross-reactive T cells may also be crucial to the cross-protection provided by rTTV-RBD-HA2 against influenza viruses. It is important to note that we used peptide pools of the H7N9 HA2, one of the immunogens in rTTV-RBD-HA2, to show the induction of HA2-specific T-cell immunity, whereas, in virus challenge studies, the rTTV-RBD-HA2-mediated protection was demonstrated against pdmH1N1 and an H3N2 virus. The usage of H3N2 for this demonstration followed the consideration that it represents a major subtype responsible for seasonal epidemics and belongs to group 2 viruses like H7N9. Aligning with the 69% sequence identity between the H7N9 HA2 and the H3N2 HA2, our preliminary analysis of T-cell epitopes has revealed that the two most reactive peptide sub-pools of the H7N9 HA2 contain at least three predicted T-cell epitopes potentially shared with the H3N2 HA2 (**Supplementary Figure 5**). This conservation is likely to account for the observed protection against the H3N2 virus.

Our study has several limitations. First, due to the lack of access to research facilities certified to conduct animal studies with SARS-CoV and MERS-CoV, we have not had the opportunity to evaluate the protective efficacy of rTTV-RBD-HA2 against these two virus targets. For SARS-CoV, the systemic levels of RBD-specific binding antibodies and neutralizing antibodies induced by RBD-HA2 through the three-dose regimen were not very high compared to results reported in previous studies of different vaccine candidates. However, the induction of T cell responses appears to be more effective (59–61). It should be noted that the effectiveness of a vaccine is determined by the combination of neutralizing antibodies and T-cell immunity. Therefore, we have reason to speculate that, with a more effective T cell response compensating for lower neutralizing antibody induction, rTTV-RBD-HA2 could achieve effective protection against SARS-CoV through a mechanism that differs from previous single-targeting SARS-CoV vaccines. Regarding MERS vaccines, two vaccine candidates have entered clinical trials: the DNA vaccine GLS-5300 and the adenovirus vector vaccine MERS001. Both vaccines use the S protein of MERS-CoV as the immunogen (62, 63). We compared our immunogenicity data on rTTV-RBD-HA2-immunized mice through the intramuscular priming-intranasal boosts regimen with the reported data from published evaluations of GLS-5300 and MERS001. While showing comparable immunogenicity to GLS-5300, in terms of binding and neutralizing antibody responses as well as cellular immune responses, rTTV-RBD-HA2 was characterized by systemic levels of MERS-CoV-specific binding and neutralizing antibodies that appear to be higher than MERS001. Although this comparison is indirect and could be confounded by variations in experimental conditions, particularly different mouse backgrounds, it could serve as initial evidence to support the potential of rTTV-RBD-HA2 to effectively provide protection against SARS-CoV and MERS-CoV infections. Additionally, due to time constraints, we did not conduct studies on the long-term efficacy of rTTV-RBD-HA2 against SARS-CoV-2 and influenza viruses. This will be part of our future investigations. Finally, our study has revealed that lung T-cell response is crucial for the cross-protectivity of rTTV-RBD-HA2 against influenza A viruses. However, we have not yet had the opportunity to examine the effect of T-cell depletion in the coronavirus setting. Exploring this direction in future studies is necessary, as it will likely provide additional evidence supporting the general importance of T cells in the efficacy of rTTV-RBD-HA2, overcoming its low effectiveness in raising neutralizing activity in the lung. Our planned future investigation would also involve understanding the functional roles of anti-HA2 antibodies. There is increasing evidence that anti-HA2 antibodies could exert their *in vivo* antiviral effects by mediating ADCC (64–66). Exploring whether the anti-HA2 antibodies raised by rTTV-RBD-HA2 follow this same theme could be beneficial.

We also acknowledge room for improving rTTV-RBD-HA2 as a universal combined vaccine targeting coronaviruses and influenza viruses. First, it is a surprise that rTTV-RBD-HA2 failed to deliver a strong pulmonary antibody response through the same regimen that directs a potent T-cell response in the lungs. Previous research with an MVA-based, SARS-CoV-2 S-expressing vaccine showed that intranasal inoculations can lead to high lung levels of S-specific IgA and IgG (44). Thus, the issue may not lie with TTV-752-1 itself. Instead, the solution may involve improving immunogen expression and/or their presentation by antigen-presenting cells. A recent structural study on the RBD-HA2 design provides a direction for such improvement by suggesting that including an HA1 component alongside the HA2 sequence could help stabilize the trimeric configuration and the protein secretion (38). Second, exploring strategies to enhance and broaden the T-cell activation ability of rTTV-RBD-HA2, e.g., complementing RBD-HA2s with immunogens comprising conserved epitopes from internal viral proteins of coronaviruses and influenza viruses, is worth considering. The central theme surrounding these explorations would be how to balance the immunogenicity, both humoral and cellular, of the multiple antigens included in the vaccine. Alternatively, comprehensive exploration of vaccination regimens, particularly the intranasal priming-intranasal boosting, which had been demonstrated to be effective for vaccinia-based SARS-CoV-2 vaccines (44, 67), may also facilitate the effort to improve the efficacy of rTTV-RBD-HA2. Nevertheless, we hope that our development of rTTV-RBD-HA2 opens a new chapter towards the ultimate mission of conquering respiratory viral infections with a single, multi-targeting vaccine.

## Data Availability

The original contributions presented in the study are included in the article/**Supplementary Material**. Further inquiries can be directed to the corresponding authors.
