# Supplementary material for: A multi-antigen vaccinia vaccine broadly protected mice against SARS-CoV-2 and influenza A virus while also targeting SARS-CoV-1 and MERS-CoV
Source: Front Immunol. 2024 Nov 28;15:1473428. doi: 10.3389/fimmu.2024.1473428 (PMC11634893; doi:10.3389/fimmu.2024.1473428)
Supplement: Supplementary file 1 [file DataSheet1.docx]

Supplementary Material

# Supplementary Figures


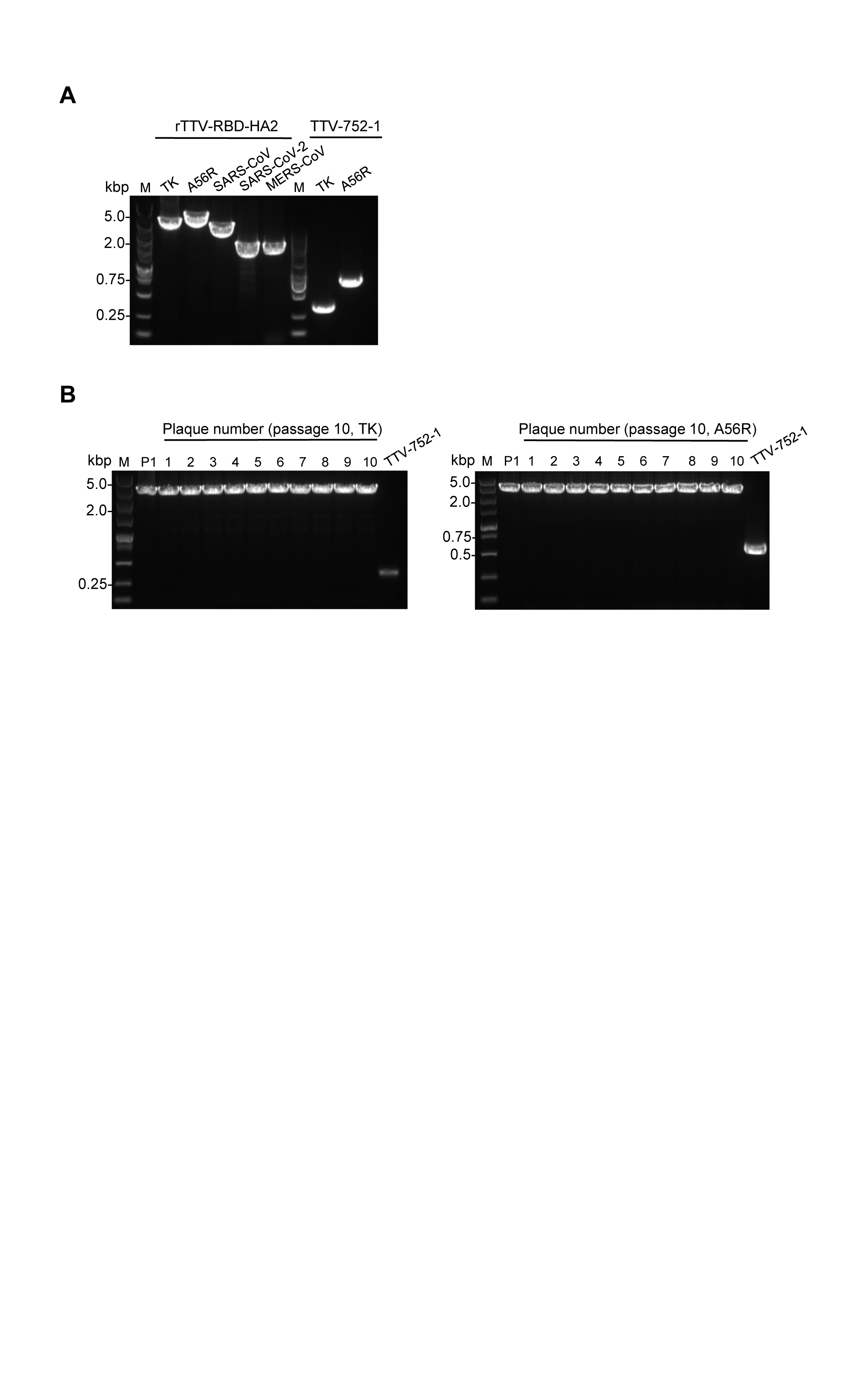


**Supplementary Figure 1.** PCR-based analyses of rTTV-RBD-HA2. (A) PCR analysis of the original rTTV-RBD-HA2 stock. Its identity and absence of parental TTV-752-1 contamination were verified by gel electrophoresis of PCR amplification product using primer pairs that matched the flanking regions of the TK or A56R gene (lanes denoted as TK and A56R) or targeted each of the corresponding RBD-HA2 genes (lanes denoted as the name of the targeted RBD-gene). The PCR reactions using TTV-752-1 as a template to amplify the TK or A56R genes were loaded as controls. (B) PCR-based validation of the genetic stability of rTTV-RBD-HA2. The passage 10 stock was subjected to a plaque assay, and ten resulting virus plaques were individually picked. These plaques, along with the parental TTV-752-1, were analyzed using PCR with primers that match the flanking sequence of TK or A56R. Gel electrophoresis was performed to detect the amplified products of insertion at the TK site (left) and the A56R site (right). Related to Figure 1D.


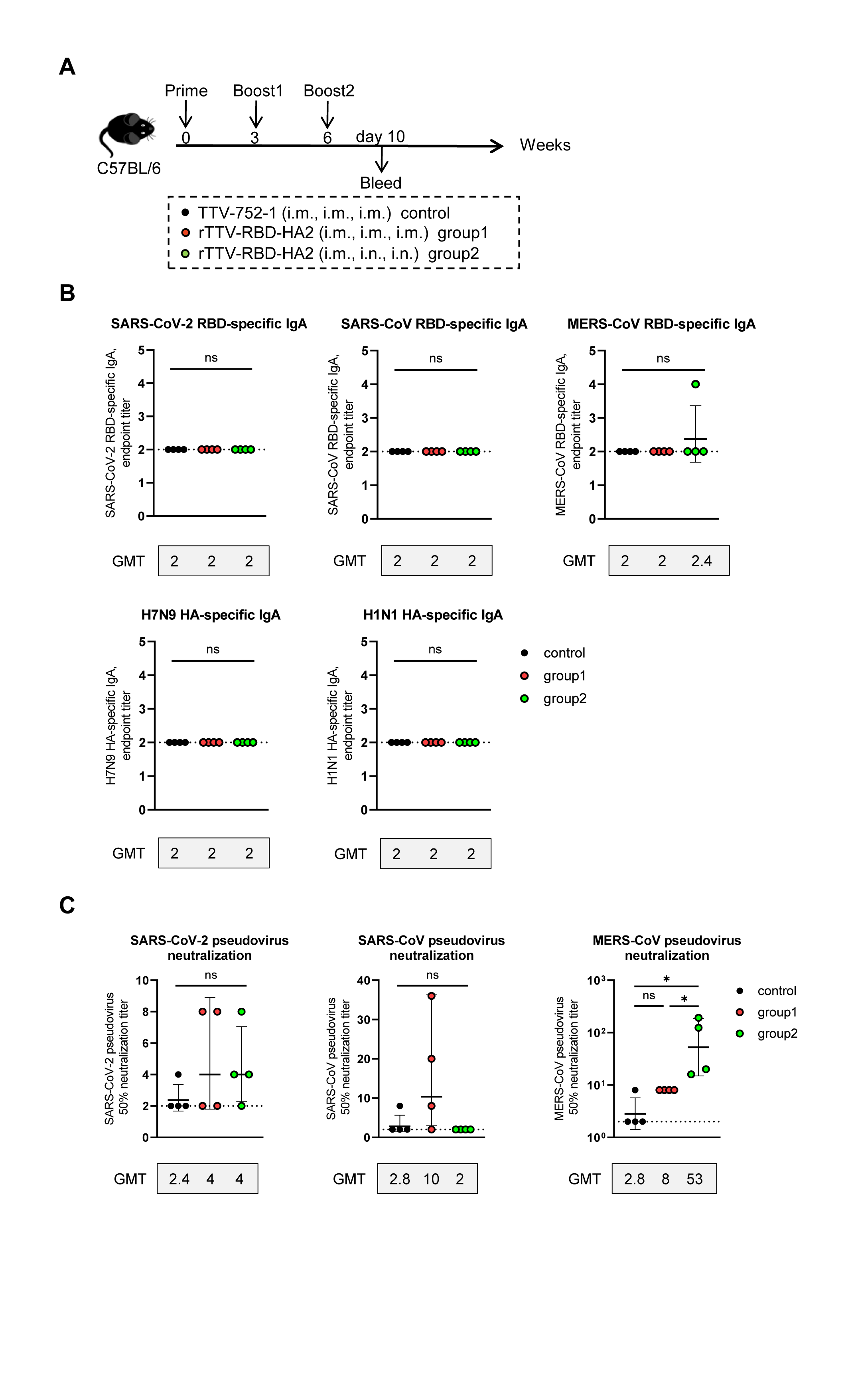


**Supplementary Figure 2.** Humoral immune responses of rTTV-RBD-HA2 in the bronchoalveolar lavage fluid (BALF) of C57BL/6 mice. The mice were immunized and sampled following the protocol in (A), which is the same as described in Figure 3A. BALF samples collected on day 10 after final immunization were analyzed for the presence of antigen-specific antibodies using ELISA (B) and pseudovirus neutralization assay (C). n=4 per group. Titers were presented as the geometric mean titer (GMT) ± geometric standard deviation (GSD). The Mann-Whitney test was used to analyze differences between experimental groups. **p*<0.05; ns, no significance. Related to Figure 2 and Figure 3.


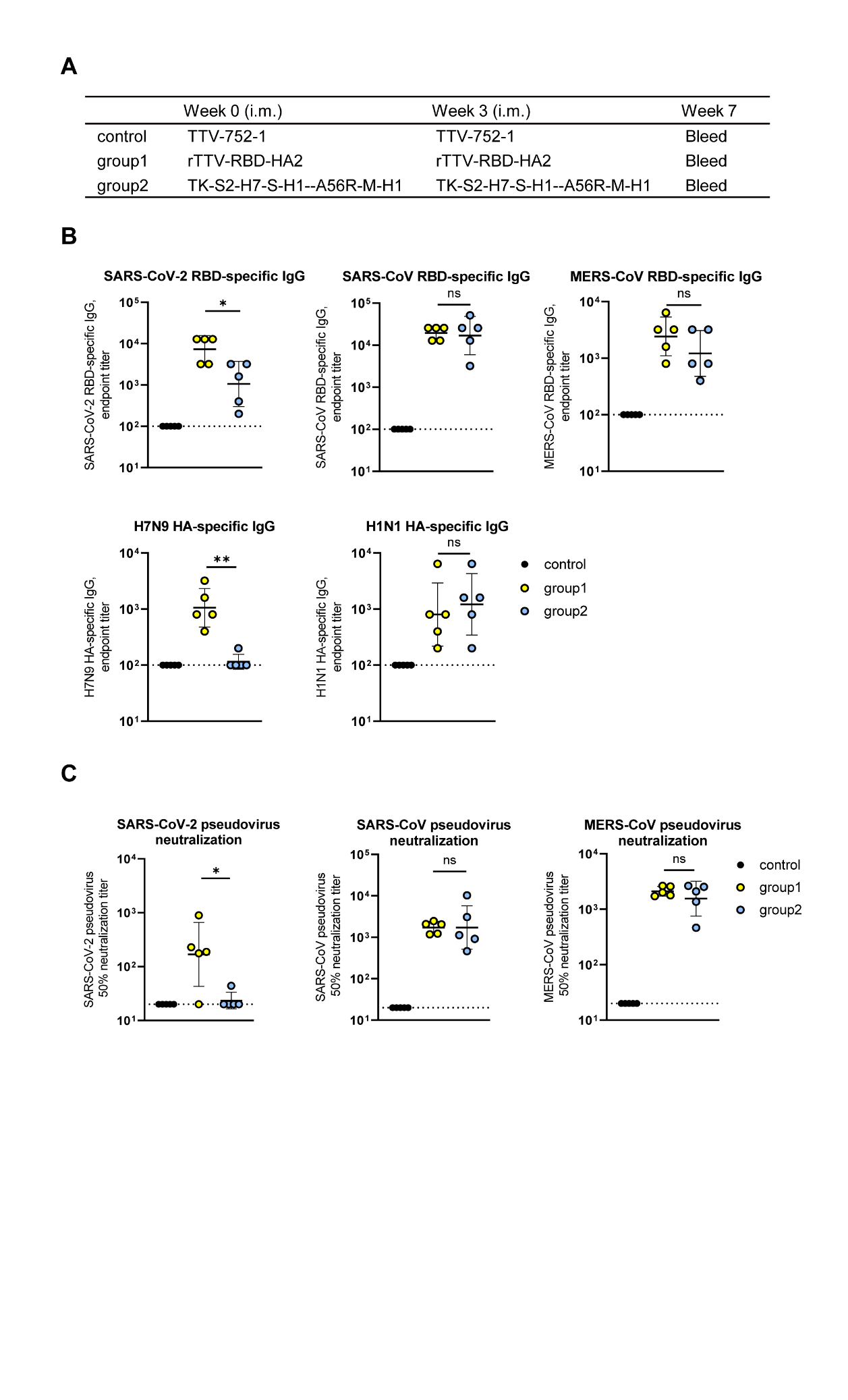


**Supplementary Figure 3.** Comparison of the immunogenicity of rTTV-RBD-HA2 and an alternative version with different RBD-HA2 arrangements. (A) Grouping of mice and the corresponding immunizations received. The dose per immunization was 1×10^7^ PFU. (B-C) Antibody responses against coronaviruses and influenza viruses analyzed at week 7 after the initial immunization. Titers were presented as the geometric mean titer (GMT) ± geometric standard deviation (GSD). n=5 per group. The Mann-Whitney test was used to analyze differences between experimental groups. **p*<0.05; ***p*<0.01; ns, no significance.


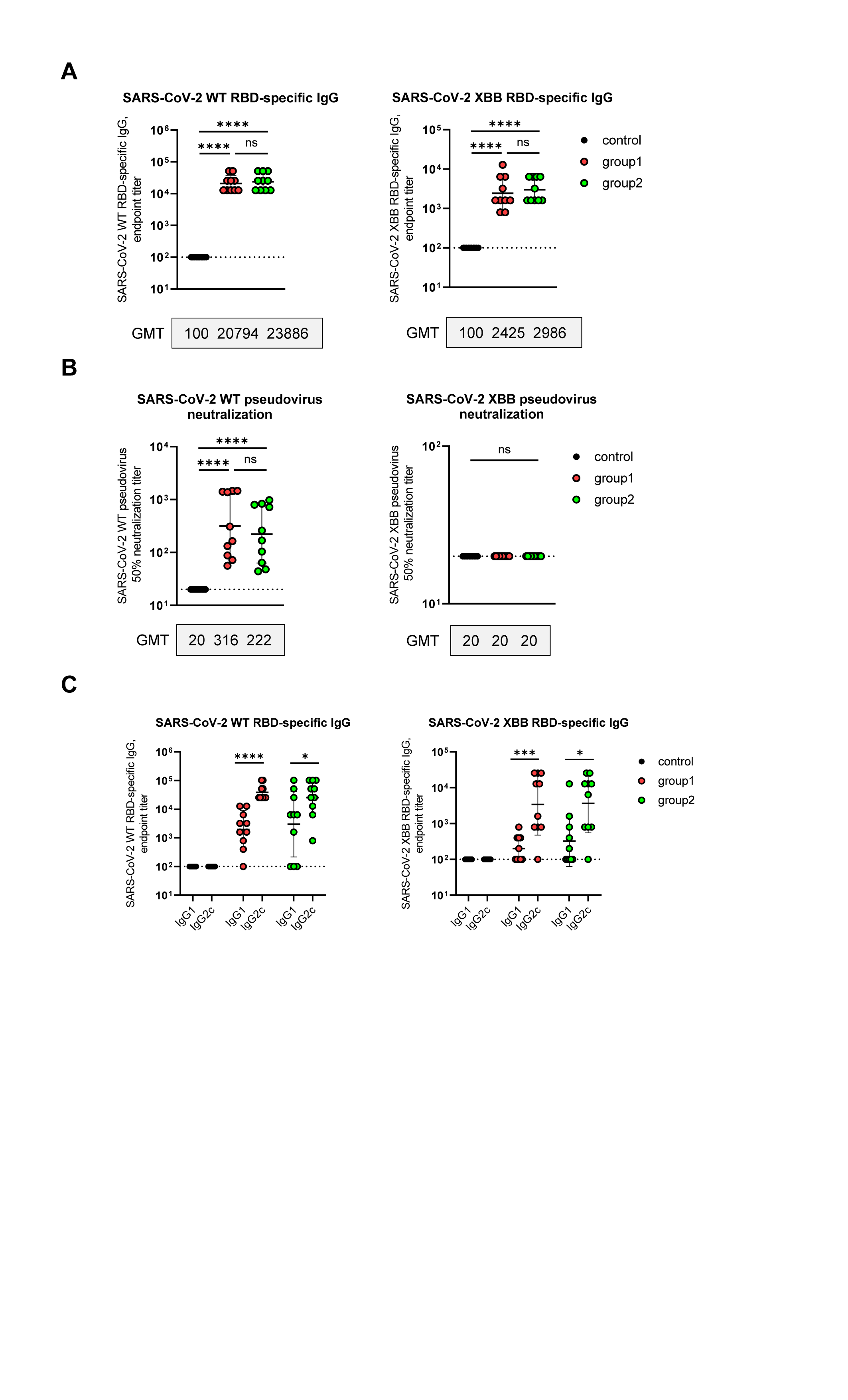


**Supplementary Figure 4.** Humoral immune responses of rTTV-RBD-HA2 in hACE2-C57BL/6 transgenic mice. Female hACE2-C57BL/6 transgenic mice were immunized following the protocol outlined in Figure 4A. Serum samples were collected at week 9 post-prime to measure binding antibody titers specific to the RBD of SARS-CoV-2 WT or the Omicron XBB subvariant using ELISA (A), as well as neutralizing titers using pseudovirus neutralization assay (B). The IgG2c and IgG1 subtype titers in the binding antibody responses were also determined using ELISA to evaluate their Th1 or Th2 bias (C). n=10 per group. Titers were presented as the geometric mean titer (GMT) ± geometric standard deviation (GSD). The Mann-Whitney test was applied when comparing experimental groups. **p*<0.05; ****p*<0.001; *****p*<0.0001; ns, no significance. Related to Figure 4.


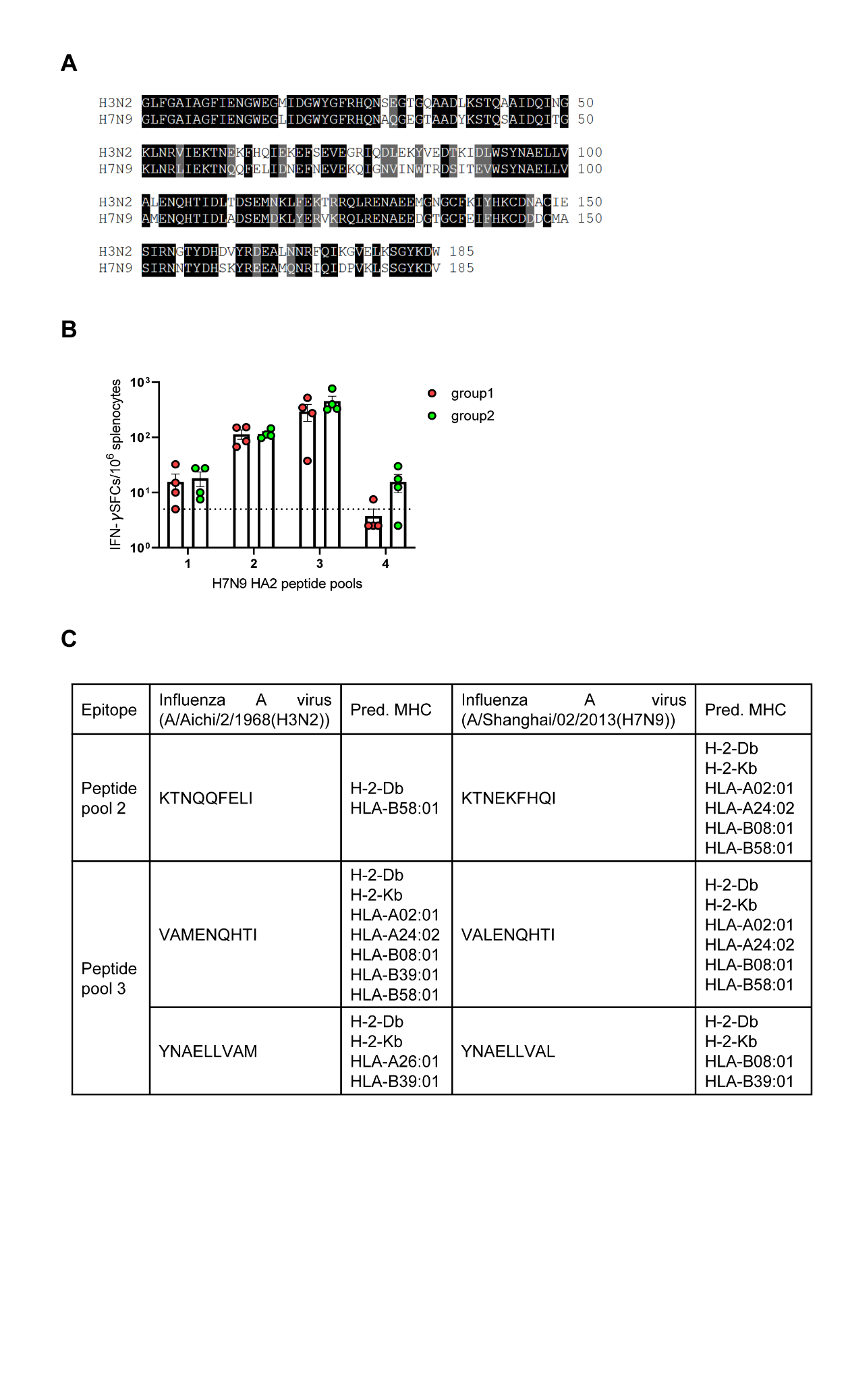


**Supplementary Figure 5.** Sequence and T-cell epitope conservation between H3N2 and H7N9 HA2s. (A) Sequence alignment of the H3N2 HA2 and the H7N9 HA2. (B) Dissection of T-cell response induced by rTTV-RBD-HA2 using peptide sub-pools. The H7N9 HA2 peptide pool was divided into 4 sub-pools and individually used to assess stimulatory activity on splenocytes isolated from rTTV-RBD-HA2-vaccinated mice on day 10 after the final immunization. Related to Figure 3. (C) Analysis of conserved T-cell epitopes between the H3N2 and the H7N9 HA2s within peptide sub-pools No. 2 and 3 assayed in (B). The analysis was performed using the *netMHCpan* software (<https://services.healthtech.dtu.dk/services/NetMHCpan-4.1/>).

# Supplementary Table

**Supplementary Table 1. Summary of vaccination regimens and challenge studies.**

| Group | Week 0 | Week 3 | Week 6 | Week 9 | Week 10 |
| --- | --- | --- | --- | --- | --- |
| Control | TTV-752-1 i.m. | TTV-752-1 i.m. | TTV-752-1 i.m. | Bleed | SARS-CoV-2 WT / XBB viruses challenge or influenza pdmH1N1 / H3N2 viruses challenge |
| Group 1 | rTTV-RBD-HA2 i.m. | rTTV-RBD-HA2 i.m. | rTTV-RBD-HA2 i.m. |  |  |
| Group 2 | rTTV-RBD-HA2 i.m. | rTTV-RBD-HA2 i.n. | rTTV-RBD-HA2 i.n. |  |  |

For the vaccination, mice were immunized with 1×10^7^ PFU of TTV-752-1 or rTTV-RBD-HA2 via the i.m. route, or were given 3×10^6^ PFU of rTTV-RBD-HA2 via the i.n. route. For challenge studies, mice were infected with 1000 PFU of SARS-CoV-2 WT or SARS-CoV-2 XBB via i.n. route, or were intranasally exposed to 10 times LD50 of influenza pdmH1N1 or H3N2 viruses.
